# UV‐Triggered Cascading Degradation of Silicone Elastomer via Self‐Fluoride Amplification

**DOI:** 10.1002/advs.202502056

**Published:** 2025-07-11

**Authors:** Yoon‐Nam Kim, Woojin Jeon, Min‐Ha Oh, Hee‐June Seo, Min Sang Kwon, Seung‐Kyun Kang

**Affiliations:** ^1^ Department of Materials Science and Engineering Seoul National University 1 Gwanak‐ro, Gwanak‐gu Seoul 08826 Republic of Korea; ^2^ Research institute of Advanced Materials (RIAM) Seoul National University 1 Gwanak‐ro, Gwanak‐gu Seoul 08826 Republic of Korea; ^3^ Nano Systems Institute SOFT Foundry Seoul National University 1 Gwanak‐ro, Gwanak‐gu Seoul 08826 Republic of Korea; ^4^ Interdisciplinary Program of Bioengineering Seoul National University 1 Gwanak‐ro Gwanak‐gu Seoul 08826 Republic of Korea

**Keywords:** fluoride self‐amplification, local UV‐Trigger, on‐demand degradation, rapid cascading degradation, silicone elastomer

## Abstract

UV‐triggered on‐demand degradable materials offering accurate and remote control of material lifetime, are emerging as vital element for applications such as self‐destructive soft robotics for hardware security, triggerable transient electronics, and data protection systems. However, previously reported UV‐initiated materials face challenges, including i) poor scalability with limited penetration depth of UV light, ii) incompatibility with UV‐blocking materials, preventing generation of reactive moieties for degradation. This study proposes a locally UV‐triggered, synergistic F^–^ self‐amplification system that enables rapid, complete, and on‐demand degradation of silicone elastomer composite, even with embedding UV‐blocking particles. This is achieved by introducing a self‐immolative F^–^ amplifying molecule (FIA) and photo‐induced F^–^ generator, diphenyliodonium hexaflurophosphate (DPI‐HFP) into the silicone elastomer which amplifies F^–^ through F^–^‐induced network of reactions. The F^–^ amplification mechanism is validated through spectroscopic, thermal and chemical analysis, and the composite shows excellent mechanical properties with elongation over 450% and low Young's modulus of ≈60 kPa. Rapid UV‐triggered degradation occurs within 60 min even with embedding 33 wt.% of magnetic particle. Furthermore, a magnetically actuated soft robot is fabricated exhibiting various motions and on‐demand degradation, underscoring the potential of this material platform for expanding the scope for UV‐triggered degradable system across various fields.

## Graphical abstract

UV‐triggered degradable materials offer proficient control of functional lifetime, while having issues with trigger efficiency. This work enhances the UV‐triggered degradation mechanism of silicone elastomer by introducing the UV‐initiated synergetic fluoride self‐amplification. The silicone composite shows excellent mechanical properties and on‐demand degradation behavior even with embedding UV hindering magnetic particles, and the remotely actuated soft robot is demonstrated.

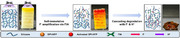

## 1 Introduction

The ability to degrade materials on‐demand or in response to specific environmental triggers, such as light,^[^ ^1^ ^]^ heat,^[^ ^2^ ^]^ pH,^[^ ^3^ ^]^ enzymes,^[^ ^4^ ^]^ and others,^[^ ^5^ ^]^ provides unprecedented control over the functional lifetime of a system, allowing users to precisely tailor degradation,^[^ ^6^ ^]^ or enable autonomous responses.^[^ ^7^ ^]^ Such programmable degradation is essential for creating smart systems with intelligent and active functional capabilities.^[^ ^1^, ^6^, ^7^, ^8^ ^]^ For instance, pH‐triggered materials are extensively applied in drug delivery for precisely targeted release of therapeutic agents,^[^ ^3a,b^ ^]^ and enzyme‐triggered or seawater‐triggered materials have been widely used for waste management minimizing environmental impact.^[^ ^4^, ^5^, ^9^ ^]^ Recent advances in transient electronics extend this concept further, allowing electronic devices to degrade on command, which proves invaluable for secure data protection,^[^ ^2^, ^6^ ^]^ and minimally invasive bio‐applications.^[^ ^10^ ^]^ Notably, trigger‐based transient systems are now being explored in soft robotics, where components can disintegrate after completing specialized missions, such as exploratory tasks, adding a new dimension to bio‐inspired robotic design.^[^ ^1^, ^8^ ^]^

Among various trigger mechanisms, UV‐initiated material systems have become prominent, offering controlled, selective wavelength utilization and the capacity to induce reactions from a distance.^[^ ^1^, ^8^, ^11^ ^]^ For instance, photo‐liable linkages such as *o*‐nitrobenzyl ester derivatives, coumarinyl esters, and azo groups have been incorporated into polymer networks to achieve UV‐triggered degradation.^[^ ^6b^ ^]^ However, materials with photo‐liable linkages were mostly limited to hydrogels with loosely cross‐linked nature, restricting their applicability in load‐bearing or structurally demanding applications. In addition to photo‐liable linkages, photo‐acid generators have been employed as an effective strategy to induce UV‐triggered degradation. In the context of transient electronics, a notable example involves cyclic poly(phthalaldehyde) (cPPA) combined with a photo‐acid generator to release acid under UV light, leading to the degradation of the polymer substrate and ultimately resulting in the disintegration of the circuit.^[^ ^2^, ^12^ ^]^ This strategy has been extended to silicone‐based elastomers like cross‐linked polydimethylsiloxane (PDMS) and Ecoflex 0030 with superior mechanical properties, incorporating photo‐generated fluoride agents (e.g., DPI‐HFP), which allow oily decomposition in soft, stretchable elastomers.^[^ ^1b^ ^]^ Owing to the superelastic properties of these materials, UV‐initiated degradation has enabled the development of lifespan‐controlled reconnaissance soft robots integrated with flexible electronics.^[^ ^1^, ^8^ ^]^

Despite the benefits, UV‐triggered degradable materials face scalability and functionality constraints. Since UV initiates reactions only on exposed surfaces, depth‐dependent degradation becomes challenging,^[^ ^1^, ^6^, ^12^ ^]^ particularly when functional particles (e.g., magnetic) obstruct UV penetration.^[^ ^1^, ^8^ ^]^ Additionally, UV‐generated radicals or reactive moieties often cleave molecular bonds one‐to‐one, requiring significant initiator concentrations to sustain effective degradation.^[^ ^1^, ^6^, ^12^ ^]^ However, excess initiators can compromise the mechanical or functional properties of the material, limiting performance optimization. Therefore, to enhance the efficacy of UV‐triggered systems, materials with amplification properties are needed—wherein degradation propagates post‐initiation, promoting sustained, self‐amplifying reactions that enable complete and efficient material disintegration.^[^ ^5d^ ^]^

Here, we propose a UV‐triggered on‐demand degradable silicone elastomer composite with rapid and complete bulk degradation with only surface UV exposure, by integration of self‐immolative fluoride ion amplifier (FIA) in the DPI‐HFP/silicone elastomer composite. The decomposition of FIA activated by initially generated F^–^ from UV exposed DPI‐HFP, sequentially drives explosive amplification and propagation of F^–^. Moreover, the H^+^ produced as a by‐product of FIA decomposition then activates DPI‐HFP in regions not directly exposed to UV, propagating F^–^ generation throughout the whole bulk composite. The spectroscopic, chemical and thermal analysis revealed the synergistic F^–^ amplification mechanism and accelerated kinetics of Si–O backbone cleavage, and the mechanical properties remained inherent to conventional silicone elastomers with excellent elongation at break and negligible hysteresis behavior. The composite showed complete degradation even with embedding magnetic particles which hinders UV penetration, by the proposed F^–^ amplification and propagation mechanism. Furthermore, the composite retained the hyperelastic properties even with embedding magnetic particles, enabling the fabrication of a largely deformable and remotely actuated soft robot with magnetic field. Various untethered motions and locomotion were demonstrated, with the ability to degrade completely upon UV‐trigger.

## 2 Results and Discussion

### 2.1 Overview and Mechanism of UV‐Triggered Cascading Silicone Elastomer Composite Degradation

#### 2.1.1 Overview of UV‐Triggered Cascading Degradation

**Figure 1:**
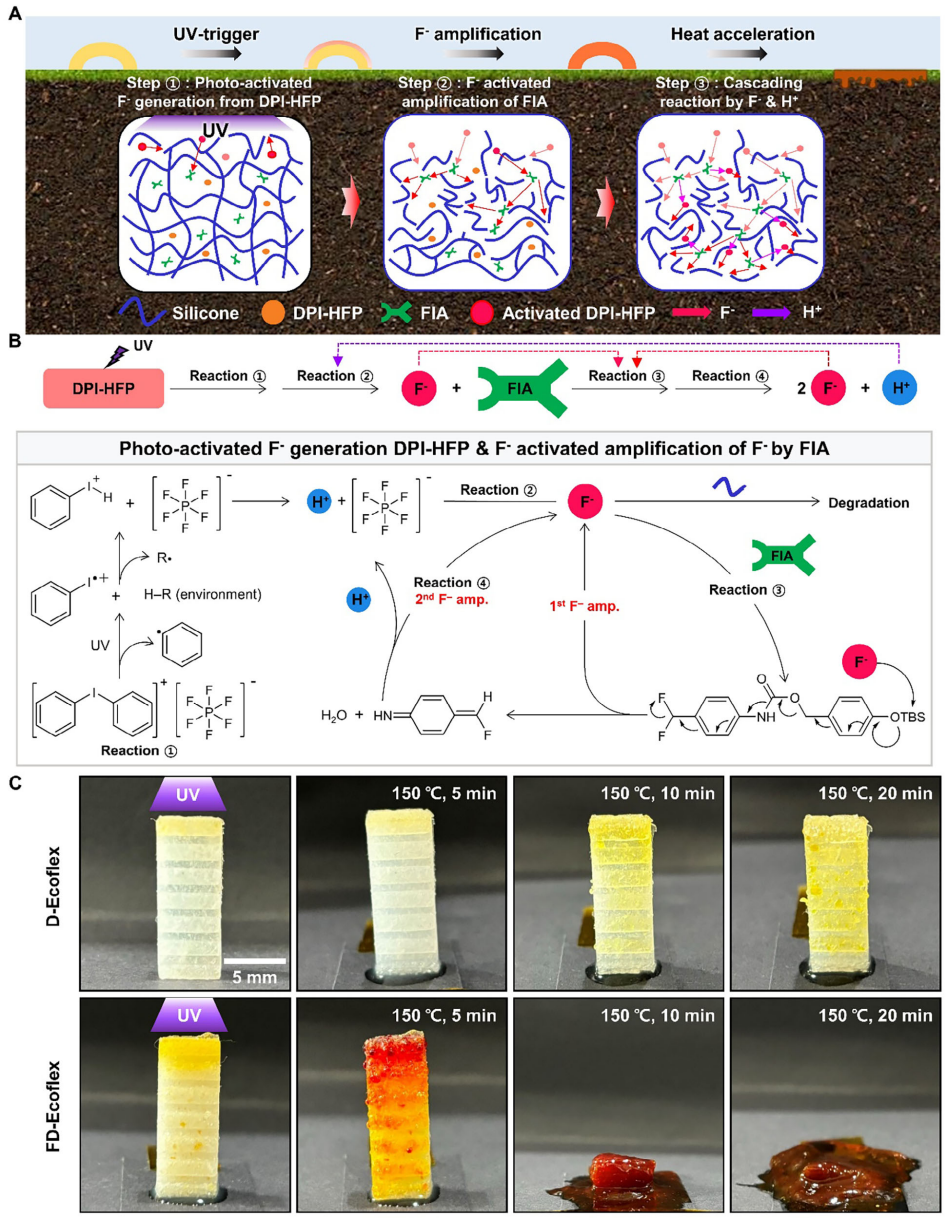
Overview of on‐demand rapid degradation of silicone elastomer by cascade Si–O cleavage reaction with fluoride ion amplifier (FIA). a) On‐demand degradation process of a FIA+DPI‐HFP+silicone (FD‐Ecoflex) composite soft robot. (Step 1) Initiated degradation with UV‐triggered F^–^ generation of surface‐residing DPI‐HFP. (Step 2) In‐depth F^–^ amplification with F^–^ diffusion and F^–^ activated amplification of FIA. (Step 3) Complete bulk degradation to liquid‐like residue and draining down the terrain with further generation of F^–^ and H^+^, activating DPI‐HFP beneath the UV‐exposed surface generating more F^–^ resulting in cascade of Si–O cleavage. b) Scheme of photo‐activated F^−^ generation from DPI‐HFP and F^–^ activated amplification of F^–^ by FIA. (Reaction 1) UV induced photolysis of diphenyliodonium ion generating H^+^. (Reaction 2) H^+^ induced decomposition of PF_6_^−^ to produce PF_5_ and F^–^. (Reaction 3) F^–^ activated decomposition of FIA molecule generating the 1^st^ amplified F^–^ from FIA. (Reaction 4) Further decomposition of 4‐(fluoromethylene)cyclohexa‐2,5‐dien‐1‐imine via water vapor producing 4‐aminobenzaldehyde, H^+^ and a 2nd amplified F^–^. c) Time‐lapse image of D‐Ecoflex (top) and FD‐Ecoflex (bottom) undergoing degradation with UV‐exposure followed by 150 °C heat exposure.

**Figure** **1** illustrates a schematic overview and the mechanism behind the accelerated degradation of a silicone elastomer composite material, triggered by UV‐induced F^–^ amplification leading to cascading cleavage reactions of Si–O bonds. Figure 1A shows the schematic overview of the accelerated degradation mechanism of the composite and a representative application scheme of a crawling robot. The composite was fabricated by adding a photo‐activated fluoride generator (DPI‐HFP, diphenyliodonium hexafluorophosphate), and a F^−^ amplifier (FIA, 4‐((tert‐butyldimethylsilyl)oxy)benzyl(4‐(difluoromethyl)phenyl)carba‐mate) to the commercial silicone‐based elastomer (Ecoflex 0030). The reaction proceeds with the following steps:

(Step 1) DPI‐HFP at the surface generates F^–^ upon initial UV‐exposure. Some of these ions cleave nearby Si–O backbones, while others activate the FIA.
(Step 2) FIA activation amplifies F^–^, which diffuse further to activate more FIA generating more F^–^.
(Step 3) This process propagates to further generation of F^–^ and H^+^, which activate DPI‐HFP beneath the UV‐exposed surface to generate additional F^–^.

These sequential F^–^ generation steps enable deeper cleavage of Si–O bonds within the composite leading to the accelerated degradation initiated by surface UV exposure.

#### 2.1.2 Mechanism of UV‐Triggered F^−^ Amplification

Figure 1B illustrates the chemistry of F^–^ amplification of FIA via UV. When UV is applied to DPI‐HFP, diphenyliodonium ion decompose and generate H^+^ by photolysis (reaction 1). H^+^ react with PF_6_^−^ to produce PF_5_ and F^–^ (reaction 2).^[^ ^13^ ^]^ In addition of FIA, F^–^ derived from DPI‐HFP with UV‐exposure attacks the silyl ether group in the FIA molecule.^[^ ^14^ ^]^ F^–^ activates decomposition of the FIA molecule producing *p*‐quinomethane, carbon dioxide, and 4‐(fluoromethylene)‐cyclohexa‐2,5‐dien‐1‐imine and F^–^ (reaction 3).^[^ ^14^ ^]^ Further decomposition of 4‐(fluoromethylene)cyclohexa‐2,5‐dien‐1‐imine via water vapor produces 4‐aminobenzaldehyde, H^+^ and a second F^–^ (reaction 4).^[^ ^14^ ^]^ Through this series of reaction steps, two F^–^ are generated by decomposition of a FIA molecule. F^–^ generated from FIA decomposition also induce the decomposition of another FIA molecule, resulting in a repetitive F^–^ amplification. H^+^ produced through reaction 4 initiates direct decomposition of hexafluorophosphate anion from DPI‐HFP by reaction 2. This enables the F^–^ generation from DPI‐HFP which was not initially activated by UV‐exposure. Therefore, when UV is applied in the presence of FIA, generation of F^–^ occurs even at the depth where UV light cannot penetrate, through cascading reaction by F^–^ and H^+^ ions. This leads to the most vigorous degradation of the silicone elastomer composite due to Si–O backbone cleavage by nucleophilic attack of F^–^ on the silicon atoms within the siloxane backbone, forming Si‐F bonds and Si–OH groups with other siloxane fragments as shown in Figure S5 (Supporting Information).^[^ ^1b^ ^]^ To verify that the degradation of the silicone elastomer was not caused mainly by acidic conditions but with F^–^, control experiments were conducted using bare silicone‐based elastomer (Ecoflex 0030). Even under strongly acidic conditions (pH 2) using HCl solution, the silicone‐based elastomer remained highly stable as shown in Figure S6 (Supporting Information). These results, consistent with previous studies, confirm that the degradation was induced by F⁻ rather than by acid.^[^ ^14b,c^ ^]^ Furthermore, the formation of Si–F bonds under the degradation conditions supports this conclusion.^[^ ^1b^ ^]^

#### 2.1.3 Effect of FIA on UV‐Triggered Silicone Elastomer Composite Degradation

Figure 1C shows the UV‐triggered degradation of the bulk Ecoflex 0030 + DPI‐HFP (20 wt.%) composite (5 mm width × 15 mm length × 2 mm thickness) with and without FIA. The composites were exposed to UV for 30 min to induce photo‐activated F^–^ generation, and heated at elevated temperature for acceleration of Si–O cleavage reaction. Heating was conducted in a convection oven to ensure a thermally uniform environment, minimizing thermal gradients throughout the composite. The temperature for acceleration was carefully chosen as 150 °C, without inducing thermal decomposition of DPI‐HFP and FIA, as shown in Figure S7 (Supporting Information). The thermal gravimetric analysis (TGA) showed the onset temperature of both DPI‐HFP and FIA's thermal decomposition of 239.2 and 239.9 °C, respectively, and the cyclic differential scanning calorimetry (DSC) analysis showed no evidence of exothermic thermal decomposition at 150 °C. The UV exposed top surface of Ecoflex 0030 + DPI‐HFP (D‐Ecoflex) exhibited a color shift toward yellow while the unexposed portion remained unchanged. Heating at 150 °C for 5 min had little effect while further heating over 10 min induced F^–^ diffusion changing the color of the composite beneath the UV‐exposed surface. Still, the degradation only occurred at the UV‐exposed top surface. UV‐exposure on Ecoflex 0030 + DPI‐HFP + FIA (FD‐Ecoflex) led to a more pronounced yellow hue suggesting increased production of F^–^ and byproducts of FIA decomposition.^[^ ^14^, ^15^ ^]^ Heating at 150 °C for 5 min accelerated the degradation of the initially UV‐exposed surface due to the higher F^–^ concentration, which increased the rate of the Si–O bond cleavage reaction. Also the whole bulk composite shifted toward yellow with F^–^ amplification reaction activated by internally diffused F^–^. The initial structure deteriorated rapidly due to a cascading reaction by F^–^ and H^+^, completely degrading within 20 min of heating. The accelerated degradation speed with FIA showed a more significant effect with increasing volume of the FD‐Ecoflex composite as shown in Figure S8 (Supporting Information). The amplification of F^–^ and cascading reaction throughout the whole bulk system enabled accelerated degradation even with larger volume. To distinguish the individual roles of UV irradiation and heat exposure in the degradation process, control experiments were conducted in which FD‐Ecoflex was subjected exclusively to either UV light or heat at 150 °C. As shown in Figure S9 (Supporting Information), FD‐Ecoflex exposed to 150 °C without prior UV treatment exhibited no visible signs of degradation even after 5 h. In contrast, when exposed only to UV light at room temperature condition, the composite showed gradual degradation, with noticeable changes after 1.5 h and complete degradation after 20 h. These results confirm that UV‐exposure serves as the primary trigger for degradation, while heat alone does not initiate the process but accelerates it when combined with UV. To further evaluate thermal stability, isothermal DSC measurements at 150 °C were conducted on both D‐Ecoflex and FD‐Ecoflex without UV pretreatment, as shown in Figure S10 (Supporting Information). Neither composite exhibited any significant thermal degradation under the isothermal condition, supporting the conclusion that both materials remain thermally stable at 150 °C in the absence of UV activation. However, the temperature limit of the thermal stability existed for both D‐Ecoflex and FD‐Ecoflex composites. Figure S11 (Supporting Information) shows the time‐lapse image of both composites at extremely high temperature (230 °C), near the thermal decomposition temperature of DPI‐HFP and FIA. Both composites showed degradation behavior without prior UV‐exposure due to decomposition of DPI‐HFP and FIA. The FD‐Ecoflex composite degraded at an increased speed possibly due to chemical reaction between FIA and DPI‐HFP at elevated temperature. While the thermal stability is limited at extremely high temperatures, the composite clearly showed UV‐triggered behavior under 150 °C condition.

Furthermore, UV‐triggered degradation of FD‐Ecoflex after 1 year storage at ambient condition was evaluated as shown in Figure S12 (Supporting Information), to ensure the long‐term stability of FIA while showing the same degradation efficiency with retained amplification mechanism upon UV‐trigger. The composite showed a dark colorimetric shift after UV‐exposure, which is a sign of byproducts formation of FIA decomposition leading to amplification of F^–^. Accordingly, the composite still showed accelerated degradation, completely degrading within 20 min of heating, ensuring the long‐term stability of FIA with unchanging amplification mechanism upon UV‐trigger.

### 2.2 Analytical Characterization of F^–^ Amplification Reaction

#### 2.2.1 Spectroscopic and Chemical Analysis of F^–^ Amplification Reaction

**Figure 2:**
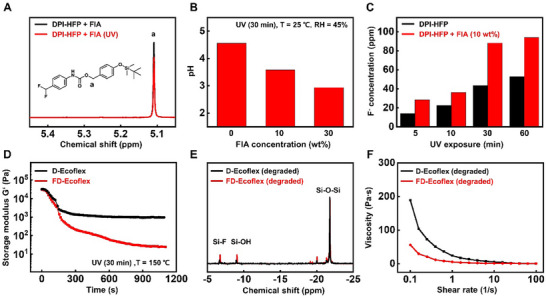
Analytical characterization of F^–^ amplification reaction a) _1_H NMR spectra of DPI‐HFP + FIA with (red) and without (black) UV‐exposure. Decrease of the benzylic proton peak (5.11 ppm) with UV‐exposure revealing the decomposition reaction of FIA activated by F^–^. b) pH variation of a mixture of DPI‐HFP with varying concentrations of FIA in deionized water after 30 min of UV‐exposure under laboratory conditions (T = 25 °C, RH = 45%). c) F^–^ concentration of DPI‐HFP (black) and DPI‐HFP with 10% FIA (red) with varying UV‐exposure time. d) Storage modulus variation of UV‐triggered D‐Ecoflex (black) and FD‐Ecoflex (red) under accelerated degradation at 150 °C. e) _29_Si NMR spectra of degraded residue of D‐Ecoflex (black) and FD‐Ecoflex (red). Si‐F bond peaks (‐7 ppm) and Si–OH bond peaks (‐9 ppm) appearing with Si–O cleavage with F^–^. (f) Viscosity of degraded residues of D‐Ecoflex (black) and FD‐Ecoflex (red) at varying shear rates.

**Figure** **2** illustrates the spectroscopic, chemical and rheological observations related to the mechanism of F^–^ amplification. In Figure 2A, the _1_H NMR spectra of FIA with DPI‐HFP after UV‐exposure support reaction 3. The peak at 5.11 ppm, corresponding to the benzylic proton, indicates the cleavage of the C‐O bond of FIA during this process. The decrease in intensity was observed due to FIA decomposition. The peak at 0.22 ppm, corresponding to the silyl ether group proton, also showed decrease in intensity with FIA decomposition as shown in Figure S13 (Supporting Information). In contrast, the _1_H NMR spectra of FIA without DPI‐HFP remain unchanged after UV‐exposure, confirming the F^–^ activated self‐immolative amplification mechanism (Figure S14, Supporting Information). Figure 2B shows the pH variation of a mixture of DPI‐HFP with varying concentrations of FIA in deionized water after 30 min of UV‐exposure under laboratory conditions (T = 25 °C, RH = 45%). DPI‐HFP without FIA exhibited a pH of 4.56 in the same condition. The addition of 10 wt.% FIA lowered the pH to 3.58, and it dropped further to 2.94 as the FIA concentration increased to 30 wt.%. The decrease in pH with increasing FIA concentration occurs as a result of the additional H^+^ formed through the hydrolysis of (fluoromethylene)cyclohexa‐2,5‐dien‐1‐imine (reaction 4), which triggers the second F^–^ amplification reaction. Figure 2C shows the F^−^ concentration of DPI‐HFP and DPI‐HFP (27 mg) with 10% FIA (3 mg) under varying UV‐exposure times. The mixtures were irradiated with UV in a closed system under laboratory conditions, then immersed in TISAB II solution (10 mL) for F^–^ concentration measurement using an ion‐selective electrode method. As UV‐exposure time increased, the F^–^ concentration also rose due to the activation of more DPI‐HFP. DPI‐HFP with 10% FIA showed higher F^–^ concentration regardless of the UV‐ exposure time, driven by the F^–^‐activated self‐immolative amplification reaction of FIA. If all 3 mg of FIA decomposes, then 12.50 ppm of F^–^ amplification is expected. However, the F^–^ concentration difference between the mixture with and without FIA exceeded 12.50 ppm across all ranges of UV‐exposure time (14.48 ppm, 5 min; 13.79 ppm, 10 min; 44.64 ppm, 30 min; 41.07 ppm, 60 min). This suggests another mechanism of F^−^ generation beyond the amplification reaction. The H^+^ produced during FIA decomposition triggers the direct breakdown of hexafluorophosphate ions from unreacted DPI‐HFP (reaction 2) leading to higher F^−^ production in the presence of FIA. The increase of UV‐exposure accelerated the amplification reaction by rising concentrations of F^−^ and H^+^ from the decomposing FIA. After 30 min of UV‐exposure, the mixture of DPI‐HFP and FIA exhibited explosive amplification surpassing 88 ppm. The rate of F^−^ generation began to plateau with further UV exposure, reaching 94 ppm after 60 min, due to complete decomposition of DPI‐HFP and FIA. Meanwhile, the variation of DPI‐HFP and FIA mass ratio also influenced the generation of F^–^. Figure S15A (Supporting Information) shows the generated F^–^ concentration with varying FIA mass under identical amount of DPI‐HFP. DPI‐HFP 200 mg (D200) with varying FIA mass DPI‐HFP 200 mg + FIA 5 mg (D200F5) and DPI‐HFP 200 mg + FIA 20 mg (D200F20) were exposed to UV with varying time. The generation of F^–^ increased with increasing FIA content, and the presence of FIA significantly accelerated the formation of F^–^ even under short UV‐exposure time (10 min). The mass of DPI‐HFP also influenced the generation of F^–^ as shown in Figure S15B (Supporting Information). The F^–^ concentration after 10 min of UV‐exposure was 35.2 ppm (D150) and 47.3 ppm (D200), respectively. Regardless of DPI‐HFP's initial mass, the presence of FIA increased the generation of F^–^ showing 54.7 ppm (D150F20) and 113.5 ppm (D200F20), respectively, after 10 min of UV‐exposure. Notably, D200 and D150F20 showed similar generation of F^–^ with UV‐exposure, suggesting the generation of F^–^ is more greatly influenced by initial FIA mass than DPI‐HFP.

#### 2.2.2 Rheological and Spectroscopic Analysis of Degradation

Figure 2D shows the change in storage modulus of UV‐triggered D‐Ecoflex and FD‐Ecoflex under accelerated degradation at 150 °C. Initially, both composites had a similar storage modulus (≈30 kPa). The storage modulus of the FD‐Ecoflex dropped ≈20 Pa corresponding to just 0.067% of its initial modulus within 20 min suggesting complete bulk degradation of the silicone elastomer composite to viscous liquid phase. In contrast, the composite without FIA reached a saturation ≈10^3^ Pa after the initial decrease ≈4 min, due to the remaining bulk composite. Figure 2E shows the _29_Si NMR spectra of degraded residue of D‐Ecoflex and FD‐Ecoflex. Both degraded residues showed Si‐F bond peaks (‐7 ppm) and Si–OH bond peaks (‐9 ppm) indicating F^–^‐induced Si–O bond cleavage. The peak integrals of Si‐F and Si–OH were normalized with the Si–O peak to compare the state of the residue. The normalized peak integrals of Si‐F and Si–OH were 0.112 and 0.107 with FIA, and 0.055 and 0.052 without FIA, respectively. This suggests harsher degradation of the composite with FIA due to amplified F^–^. The _29_Si NMR spectra analysis was also conducted on the degraded residues of the un‐crosslinked linear PDMS (M.W 4800) with and without FIA to verify the harsher degradation, and the Si–OH and Si‐F peak integrals showed higher value with FIA as shown in Figure S16 (Supporting Information).

The F^–^‐induced Si–O bond cleavage was also verified by comparing the F^–^ concentration from UV‐triggered DPI‐HFP (200 mg) and FIA (20 mg) with F^–^ concentration from degraded FD‐Ecoflex consisted with the same amount of DPI‐HFP and FIA. Figure S17A (Supporting Information) shows the comparison of F^–^ concentration generated with UV‐exposure from DPI‐HFP (160.4 ppm), DPI‐HFP with FIA (324.8 ppm) and F^–^ concentration within the degraded residue of FD‐Ecoflex (59.5 ppm). To quantify the residual F^–^ in the degradation residue, the degraded residue was fully dissolved in hexane and subjected to multiple work‐up steps with deionized water as shown in Figure S17B (Supporting Information). The work‐up process was repeated until no further F^–^ were detected in the aqueous phase. In systems where DPI‐HFP and FIA are incorporated into a silicone elastomer matrix (FD‐Ecoflex), the F^–^ concentration recovered was significantly reduced (−82%) after degradation. This reduction suggests the consumption of F^–^ during the degradation process of the silicone elastomer, producing Si‐F groups from Si–O scission.

Furthermore, _1_H NMR and _19_F NMR spectra analysis were conducted to clarify the byproducts of FD‐Ecoflex degradation residue as shown in Figure S18 (Supporting Information). The _1_H NMR spectra revealed that the most intense proton signals originated from the methyl groups of degraded siloxane, indicating that siloxane fragments are the primary decomposition product.^[^ ^13b^ ^]^ Additionally, residual DPI‐HFP was still detected after decomposition, and iodobenzene—known as a major photodegradation product of DPI‐HFP—was also identified. This finding is consistent with a previous report.^[^ ^13a^ ^]^ In the case of FIA, it was completely consumed, and 4‐aminobenzoate was identified as the decomposition product. This result aligns with previous literature on FIA derivatives.^[^ ^13c^ ^]^ Based on the _19_F NMR analysis, PO_3_F^2–^ and PO_2_F_2_⁻, which are derived from the decomposition of PF_6_⁻, were identified as major fluorine‐containing byproducts, showing relatively low toxicity also used in commercial dentrifrices.^[^ ^13c,d^ ^]^ Additionally, SiF_6_^2^⁻, formed through the degradation of the silicone elastomer, was also observed as a major product.^[^ ^13c^ ^]^ Notably, HF was not detected in the _19_F NMR spectrum, suggesting that most of the generated HF was consumed during the degradation of the silicone elastomer during nucleophilic attack of F^–^ on siloxane resulting in Si–O scission consistent with the _29_Si NMR analysis. While byproducts such as PO_2_F_2_⁻ contains a possibility of forming additional HF in certain conditions such as extremely low pH, the HF detected in the residue was negligible in our condition.^[^ ^13e^ ^]^

Figure 2F compares the viscosity of degraded composites at varying shear rates. Both composites exhibit shear thinning behavior, attributed to chain disentanglement.^[^ ^16^ ^]^ The composite containing FIA shows a viscosity of 5.5 Pa∙s at a shear rate of 1 rad s^−1^, while the composite without FIA has a viscosity of 24.1 Pa∙s. Across all shear rates, the degraded residue of FD‐Ecoflex consistently demonstrates lower viscosity, implying lower molecular weight of the residue, according to the Mark‐Houwink equation,

(1) $$\eta =K\;\cdot \;M^{a}$$

where η is the viscosity, M is the molecular weight, and K, ɑ are constants specific to the polymer‐solvent system.^[^ ^17a^ ^]^ Given that the constants K, ɑ can be assumed to be the same for both samples under identical conditions, the lower viscosity of the FD‐Ecoflex residue suggests lower molecular weight of the residue, likely due to more aggressive degradation.^[^ ^17^ ^]^

### 2.3 Kinetic and Thermodynamic Analysis of FD‐Ecoflex Degradation

**Figure 3:**
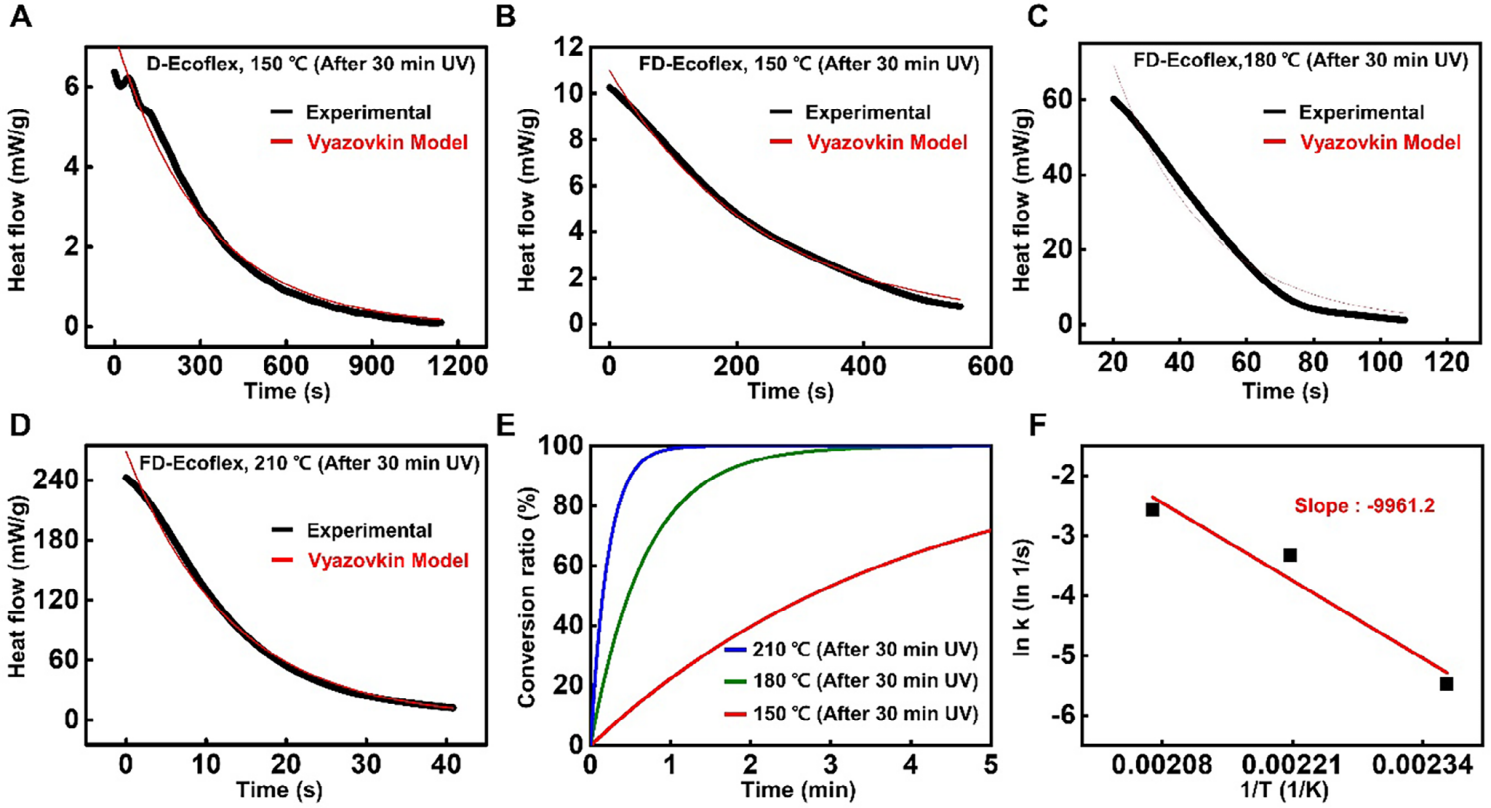
Kinetic and thermodynamic studies regarding FD‐Ecoflex degradation. DSC Heat flow analysis for the degradation reaction at 150 °C after 30 min UV‐exposure of the a) D‐Ecoflex and b) FD‐Ecoflex (black, experiment; red, Vyazovkin model fitting). DSC heat flow analysis for the degradation reaction of FD‐Ecoflex after 30 min UV at c) 180 °C and d) 210 °C (black, experiment; red, Vyazovkin model fitting). e) Extent of phase conversion (α) of FD‐Ecoflex from a solid elastomer to a liquid‐like phase over time during degradation at various temperatures (150 °C, red; 180 °C, green; 210 °C, blue). f) Arrhenius plot of the FD‐Ecoflex degradation reaction plotted with k values at various temperatures (0.0042, 150 °C; 0.0245, 180 °C; 0.0766, 210 °C), and the slope indicating the activation energy of the Si–O cleavage (9903.2 (slope) × 8.314 kJ mol^−1^ = 82.33 kJ mol^−1^).

**Figure** **3** presents the kinetics and the thermodynamics of the degradation mechanism regarding the FD‐Ecoflex composite. DSC analysis provides the kinetics and the activation energy of the degradation process. Figure 3A,B shows the heat flow over time for the degradation reaction of the D‐Ecoflex and FD‐Ecoflex composite under constant temperature (150 °C) after 30 min UV‐exposure. Both showed an exothermic heat flow owing to the exothermic Si–O cleavage reaction.^[^ ^18^ ^]^ The heat flow for the D‐Ecoflex composite lasted for≈1100 s with initial value of≈6 mW g^−1^. The accelerated heat flow was observed for FD‐Ecoflex with higher initial value of≈10 mW g^−1^ and the reaction ending at≈550 s. This demonstrates that the acceleration of the F^–^ generation within FD‐Ecoflex increases the overall concentration of reactant (F^–^), thereby kinetically accelerating the reaction. The heat flow for the degradation reaction of FD‐Ecoflex were analyzed with increased temperature of 180 °C (Figure 3C), and 210 °C (Figure 3D), respectively. The initial heat flow increased accordingly with higher temperature showing the value of≈60 mW g^−1^ (180 °C) and≈240 mW g^−1^ (210 °C), and the reaction terminating faster at≈100 s (180 °C) and≈40 s (210 °C), respectively.

Figure 3E illustrates the extent of phase conversion (α) of FD‐Ecoflex from a solid elastomer to a liquid‐like phase over time during degradation at various temperatures (150, 180, 210 °C). The value of α is determined from heat flow obtained through DSC analysis, specifically by measuring the heat released during the exothermic Si–O cleavage.^[^ ^19^ ^]^ It is calculated with the equation:

(2) $$\alpha =\frac{\Delta H_{t}}{\Delta H_{total}}$$

where ∆H_t_ is the heat released up to time t, and ∆H_total_ is the total heat released throughout the degradation process. The conversion rate increased with temperature and the FD‐Ecoflex composite fully degraded in ≈1 min at 210 °C. This highlights the accelerated reaction rate associated with higher temperatures in accordance with the Arrhenius equation.^[^ ^20^ ^]^ The reaction coefficient (*k*) was obtained with the following equation:

(3) $$\alpha =1-e^{-kt}$$

assuming Vyazovkin model.^[^ ^19^ ^]^ The value *k* for various temperatures were acquired and plotted as the Arrhenius plot (0.0042, 150 °C; 0.0245, 180 °C; 0.0766, 210 °C) (Figure 3F), increasing with temperature showing accelerated degradation due to sufficient energy for overcoming the activation energy barrier of the Si–O cleavage. The activation energy was acquired from the Arrhenius equation with the value of 82.33 kJ mol^−1^, which is comparable with the previously calculated activation energy of Si–O bond cleavage from SiO_2_ by F^–^ in HF solution via extended Hückel calculation (77.19 kJ mol^−1^).^[^ ^21^ ^]^ The density functional theory (DFT) calculation of the 1,5‐dimethoxy‐1,1,3,3,5,5‐hexamethyltrisiloxane molecule similar to short chained PDMS were also performed as shown in Figure S19 (Supporting Information) to verify the exothermic Si–O cleavage reaction and to confirm that the Si–O bond cleavage reaction still remains as the rate determining step of degradation while FIA was introduced into the system. In addition, DSC analysis for the silicone elastomer composite with reduced FIA content (0.5 wt.%) was conducted in the same experimental setup, to clarify the influence of FIA on degradation thermodynamics as shown in Figure S20 (Supporting Information). The reaction coefficient (*k*) for each temperature conditions were obtained in the same manner, and showed the value of 0.0027 (150 °C), 0.0103 (180 °C) and 0.0213 (210 °C), respectively. The k values for all temperature condition was reduced, suggesting the impeded degradation with lower FIA content. Consequently, the extent of phase conversion was also impeded for all temperature ranges, clarifying the influence of FIA ratio on degradation kinetics.

Furthermore, the total energy consumption required for complete degradation was compared between D‐Ecoflex and FD‐Ecoflex to show the improved degradation efficiency as shown in Figure S21 (Supporting Information). The UV‐lamp (VL‐215C) with a power of 1.78 mW cm^−2^ and a manually fabricated micro‐heater was used for precise calculation of energy consumed during degradation. The total energy required for complete degradation was greatly reduced from 16.4 kJ (D‐Ecoflex) to 3.4 kJ (FD‐Ecoflex), with the introduction of synergetic amplification of F^–^. Moreover, the energy consumption for complete degradation was greatly reduced with the use of a focused UV laser, which possesses significantly higher power density (125 mW cm^−2^) compared to UV lamp. Figure S22 (Supporting Information) shows the degradation behavior and energy consumption for complete degradation using focused UV laser. FD‐Ecoflex showed complete bulk degradation with focused UV laser, while bare Ecoflex and D‐Ecoflex was not possible to degrade completely with only focused UV laser. Total energy consumption for complete degradation with focused UV laser was greatly reduced to 0.5 kJ for bulk FD‐Ecoflex sample, suggesting the possibility of improving the energy efficiency of degradation for FD‐Ecoflex with cascading degradation property.

### 2.4 Mechanical Properties of FD‐Ecoflex Composite

**Figure 4:**
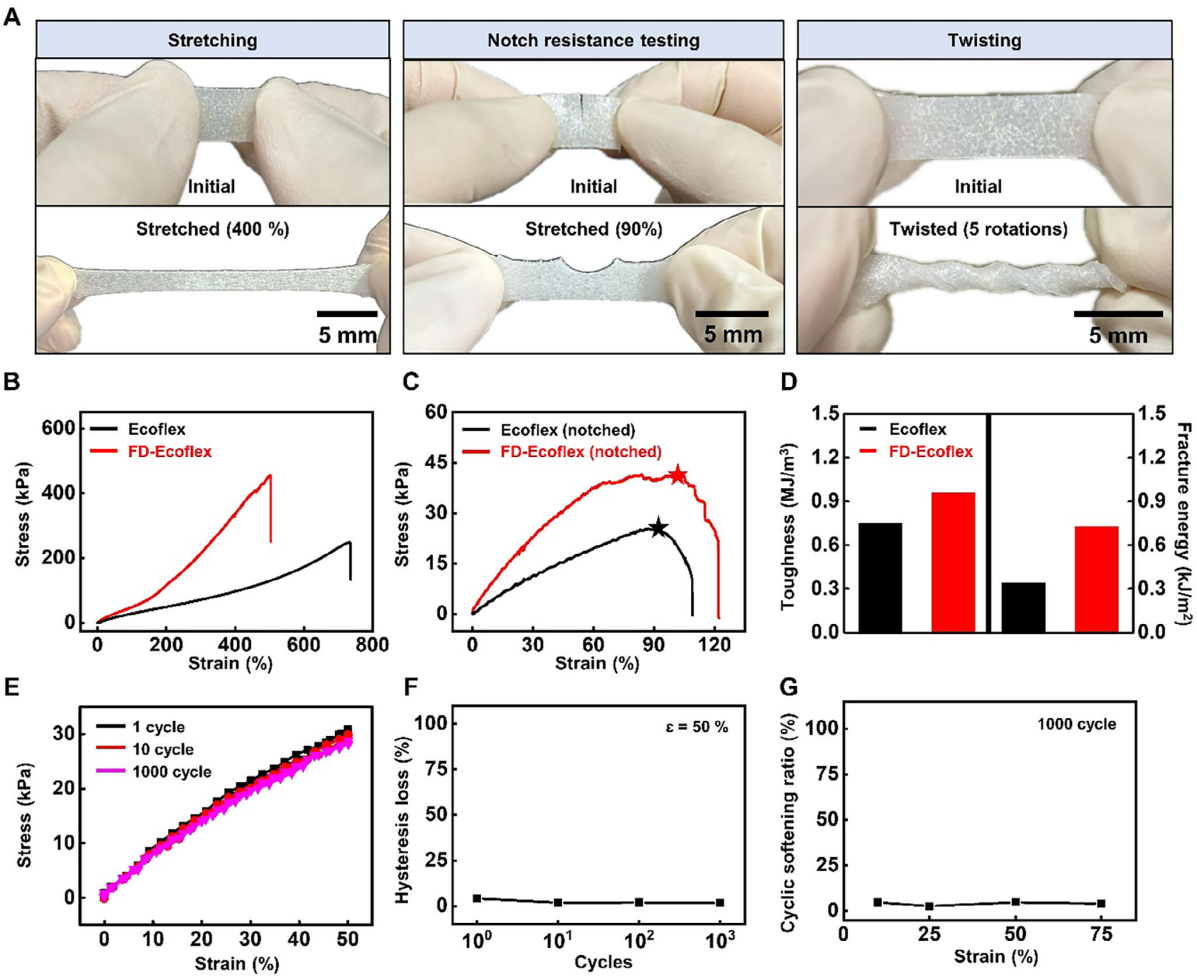
Mechanical properties of the FD‐Ecoflex composite. a) Various deformations (stretching ∼400%, left; 2.5 mm notch resistance testing≈90%, middle; 5 rotation twisting, right) of the FD‐Ecoflex composite without any noticeable plastic deformations. b) Tensile stress‐strain curve of the FD‐Ecoflex (red) and bare Ecoflex (black) with a sample dimension of 30 mm × 5 mm × 1 mm (length × width × thickness), showing elastic modulus (48.8 kPa, FD‐Ecoflex; 28.8 kPa, bare Ecoflex) and elongation at break (453%, FD‐Ecoflex; 736%, bare Ecoflex). c) Stress‐stretch curve of the notched samples of FD‐Ecoflex (red) and bare Ecoflex (black) with a width of 10 mm, thickness of 1 mm, height of 20 mm, and a notch of 5 mm. Critical stretch of crack propagation showing 104% for FD‐Ecoflex (red star) and 91% for bare Ecoflex (black star), respectively. d) Toughness and fracture energy of the FD‐Ecoflex (red) and bare Ecoflex (black) (0.96 vs. 0.75 MJ m^−3^ and 0.73 vs. 0.34 kJ m^−2^, respectively). e) Cyclic tensile stress‐strain curve of the FD‐ecoflex with 50% strain and 20 mm min^−1^ strain rate. (1 cycle, black; 10 cycle, red; 1000 cycle, magenta). f) Hysteresis loss of the FD‐ecoflex with various stretch cycles with 50% strain. (g) Cyclic softening ratio of the FD‐Ecoflex after 1000 cycles with varying strain.

**Figure** **4** presents the mechanical properties of the FD‐Ecoflex composite. Figure 4A displays the various deformations of the FD‐Ecoflex composite including stretching, notch resistance testing, and twisting. The composite returned to its original state without any plastic deformation even after 400% stretching and twisting by making 5 rotations. Also the composite with 2.5 mm notch showed no advancement of the crack with≈90% stretching. Figure 4B shows the tensile stress‐strain curve of the FD‐Ecoflex and bare Ecoflex with a sample dimension of 30 mm × 5 mm × 1 mm (length × width × thickness). The elastic modulus for the FD‐ecoflex and bare Ecoflex were 48.8 and 28.8 kPa, and the elongation at break were 453% and 736%, respectively. The incorporation of FIA increased the elastic modulus through the filler reinforcement effect,^[^ ^22^ ^]^ while simultaneously decreasing elongation due to stress concentration at the silicone‐filler interface.^[^ ^23^ ^]^ Still, the FD‐Ecoflex composite showed a low range modulus of kPa scale and high elongation at break over 400%, applicable for soft and stretchable substrate or frame material for soft robotics.^[^ ^24^ ^]^ The stress‐stretch curve of the notched samples of FD‐Ecoflex and bare Ecoflex with a width of 10 mm, a thickness of 1 mm, height of 20 mm, and a notch of 5 mm were obtained (Figure 4C). The critical stretch of crack propagation was 104% and 91% for the FD‐Ecoflex and bare Ecoflex, respectively. Toughness and fracture energy of FD‐Ecoflex were higher than those of bare Ecoflex (0.96 vs. 0.75 MJ m^−3^ and 0.73 vs. 0.34 kJ m^−2^, respectively; Figure 4D). Here the toughness was acquired from the area beneath the stress‐strain curve (Figure 4B). The fracture energy was derived from the stress‐stretch curve of the notched sample (Figure 4C) by Rivlin and Thomas equation.^[^ ^25^ ^]^ Figure 4E shows the cyclic tensile stress‐strain curve of the FD‐ecoflex at a uniaxial strain of 50% with the strain rate of 20 mm min^−1^. The FD‐ecoflex exhibited negligible elastic hysteresis showing 1.56% hysteresis loss at 1000 cycles (Figure 4F). Figure 4G shows the cyclic softening ratio of the FD‐Ecoflex composite after 1000 cycles with varying strain. Due to the excellent property of the base elastomer (Ecoflex‐0030), the softening behavior of the FD‐Ecoflex with 1000 cyclic strain was negligible up to 75% strain verifying the wide elastic range of the composite suitable for soft robotic applications.^[^ ^26^ ^]^

### 2.5 UV‐Triggered Degradation in Magnetically Actuated Soft Robots

**Figure 5:**
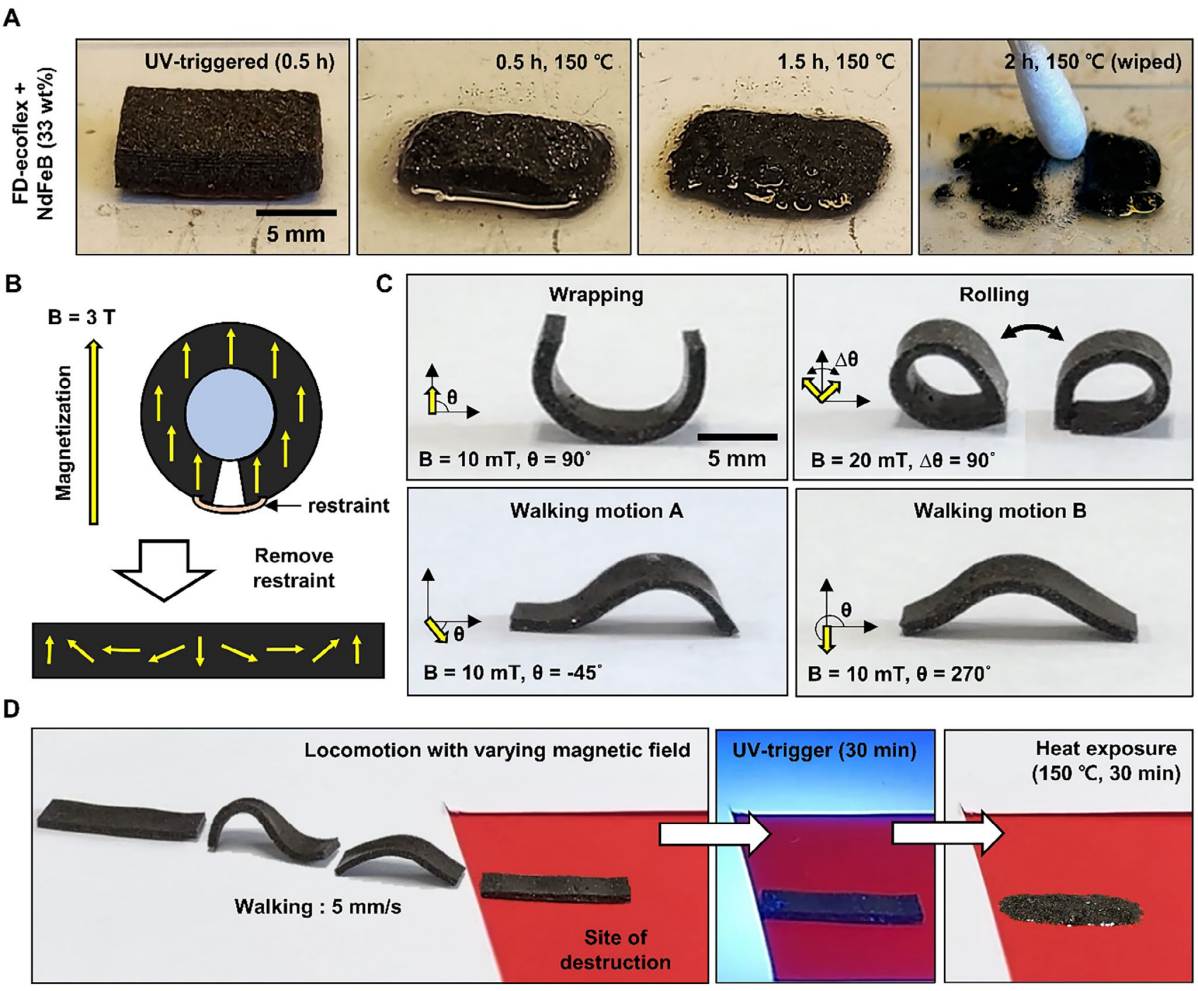
Application of UV‐triggered degradation in magnetically actuated soft robots. a) Time lapse image of on‐demand degradation of the FD‐Ecoflex composite embedded with magnetic particle (FD‐Ecoflex + NdFeB 33 wt.%). Heating for 30 min (150 °C) leading to destruction of initial shape and wiped out with complete degradation after 120 min heating (150 °C). b) Scheme of the fabrication and magnetization strategy for triggering degradable soft robot. Rectangular shaped body wrapped around a cylindrical frame and magnetized with magnetic field (3 T) with VSM acquiring programmed non‐uniform magnetization profile. c) Various actuation motions actuated with varying magnetic field (Wrapping, top left, 10 mT, 90 degrees; Rolling, top right, 20 mT, 45 degrees to 135 degrees; walking motion A, bottom left, 10 mT, ‐45 degrees; walking motion B, bottom right, 10 mT, ‐90 degrees). d) Robotic demonstration of locomotion to site of destruction. Robot showing the velocity of 5 mm s^−1^ (Body length s^−1^ = 0.25) and fully degrading after 30 min heat exposure (150 °C) after 30 min UV‐exposure.

Incorporation of FIA induce amplification and propagation of F^–^ throughout the whole composite with self‐immolative F^–^ generation, enabling UV‐triggered degradation even with the introduction of other functional fillers such as magnetic particles into the composite functionalizing remote robotic actuation which could hinder the penetration of UV.^[^ ^8^, ^27^ ^]^ **Figure** **5** demonstrates the UV‐triggered degradation of the FD‐Ecoflex composite blended with magnetic particle, and its soft robotic application with untethered actuation. Figure 5A shows the on‐demand degradation of the FD‐Ecoflex composite embedded with magnetic particle (FD‐Ecoflex + NdFeB 33 wt.%). The composite appeared in a blackish hue due to the natural color of NdFeB. After 30 min of UV‐exposure, heating the composite for 30 min (150 °C) led to destruction of the initial shape of the composite. With further heating for 60 min, the composite completely lost its shape. The degraded residue could be wiped out completely after heating 120 min showing complete phase transition from solid elastomer to liquid. Figure 5B illustrates the fabrication and magnetization strategy for triggerable soft robot. A resin with composition of Ecoflex 0030: DPI‐HFP: FIA: NdFeB = 10: 2: 0.2: 3 (w/w) was poured into a rectangular shaped mold and the robot body with 20 mm × 4 mm × 1 mm (length × width × thickness) was fabricated. The robot body was wrapped around a cylindrical frame and magnetized with strong magnetic field (3 T) using vibrating sample magnetometer (VSM) to acquire the programmed non‐uniform magnetization profile for various actuating motions.^[^ ^8^, ^28^ ^]^

Figure 5C displays the various motions (wrapping, rolling, walking) actuated with varying magnetic field. The robot demonstrated a wrapping motion with magnetic field of 10 mT perpendicular to the ground surface. Increasing the magnetic field to 20 mT completely wrapped the robot into a round shape and the robot showed a rolling motion by shifting the direction of the magnetic field from 45˚ to 135˚ (x‐z plane) as shown in Supplementary video. The robot also demonstrated a walking motion with alternating walking motion A and B, while motion A and motion B were exhibited through 10 mT magnetic field with −45˚ (x‐z plane, motion A) and ‐90˚ (x‐z plane, motion B), respectively. Figure 5D demonstrates the situation where the robot locomotes to the site of destruction remotely to undergo triggered degradation. The robot showed the velocity of 5 mm s^−1^ (Body length s^−1^ = 0.25) comparable to previous soft robots.^[^ ^29^ ^]^ After reaching the site of destruction, the robot was triggered with 30 min UV and fully degraded into an oily liquid state with 30 min heating (150 °C), precluding any possibility of recovery.

## 3 Conclusion

The proposed FD‐Ecoflex system, utilizing UV‐triggered F^–^ generation and a synergetic self‐immolative F^–^ amplification reaction, introduces an innovative mechanism for the rapid and complete on‐demand degradation of bulk silicone‐based elastomers, while maintaining their excellent mechanical properties. When F^−^ is generated by UV‐triggered DPI‐HFP, the FIA molecule sequentially drives explosive amplification and propagation of F^–^. The H^+^ produced as a by‐product of FIA decomposition then activates DPI‐HFP in regions not directly exposed to UV, propagating F^–^ generation. This mechanism effectively overcomes the limitation of UV penetration caused by the presence of magnetic particles, which otherwise impedes complete bulk degradation. By integrating magnetic particles while preserving the mechanical properties of the silicone elastomer, a remotely actuated soft robot with on‐demand degradability was successfully demonstrated. While the synthesis methods and yield of the FIA molecule need further development for scale‐up and mass production, the acceleration of the UV‐triggered degradation mechanism offers promising potential. This approach enables the future development of instant self‐destructive systems that maintain stretchability, applicable in fields requiring immediate transience, such as security robots, military drones, actively detachable soft silicone adhesives, and security IC chips.

## 4 Experimental Section

### Synthesis of Fluoride Ion Amplifier (FIA)

4‐((tert‐Butyldimethylsilyl)oxy)benzyl (4‐(difluoromethyl)phenyl)carbamate (FIA) was synthesized through a three‐step reaction, with (4‐((tert‐butyldimethylsilyl)oxy)‐phenyl)methanol, used in the third step, being prepared in a one‐step reaction. Detailed synthesis procedures and characterization are provided in the Supporting Information.

### Fabrication of FD‐Ecoflex Composite

Diphenyliodonium hexafluorophosphate (DPI‐HFP; TCI, Japan) was added to Ecoflex 0030 prepolymer (Smooth‐On, USA) with a mass ratio of 20 wt.%, placed in a mixing container and evenly mixed with a mixer (ARE‐310, THINKY, Japan) using the planetary centrifugal mixing mode, at a rotation speed of 2000 rpm for 2 min. Thereafter, the synthesized FIA molecule was added to the mixture with a mass ratio of 2 wt.% and evenly mixed with a mixer following the same procedure. The mixture was then poured into a 3D printed mold fabricated with ABS and cured in an oven at 70 °C for 20 min.

### Analysis of FIA Molecule Decomposition and F^−^ Amplification

The UV‐trigger was induced by UV lamp (VL‐215C, Vilber Lourmat, France) consisted with 2×15 W tubes of 1780 µm cm^−3^ intensity (254 nm) and exposed at a distance of 10 cm for all samples. The ^1^H NMR data were obtained with 600 MHz high resolution NMR (Avance III HD, Bruker, Germany) using acetone as a solvent, for decomposition analysis of FIA. The H^+^ and F^–^ ion concentration was obtained by using a selective ion method with an all‐in‐one titrator (OrionStarT940, Thermo Scientific, USA). TISAB II was used as a solvent for F^–^ concentration analysis and Deionized water (DI) was used for H^+^ ion concentration analysis. While DI shows relatively low pK_a_≈3.2 with HF, the pH value indicates the trend of the H^+^ concentration difference following the equation:

(4) $$\mathrm{pH}=-\frac{1}{2}\;\left(pK_{a}+\log{\left[HF\right]}_{0}\right)$$

as only HF is considered in all cases.

### Degradation Analysis of FD‐Ecoflex Composite

To analyze the degradation process of the FD‐Ecoflex composite, a disk shaped composite with and without FIA was fabricated with a 3D printed mold (diameter 10 mm, thickness 5 mm). UV‐exposure was conducted for 30 min and the storage modulus change were analyzed using a rheometer (MCR302e, Anton Paar, Austria) with 150 °C isothermal time sweep condition with 1% shear strain at 10 rad s^−1^. The degradation kinetics were also analyzed using differential scanning calorimeter (Discovery DSC 2500, TA Instrument, USA) with 150 °C isothermal time sweep condition. The ^29^Si NMR data of the degraded residues were analyzed with 600 MHz high resolution NMR (Avance III HD, Bruker, Germany) using tetrahydrofuran as a solvent, and viscosity data with advanced rheometric expansion system (ARES, Rheometric Scientific, UK).

### Density Functional Theory Calculations

Geometry optimizations, single‐point energy calculations, and transition state calculations were all performed using the Gaussian 16 program package with the dispersion (D3)‐corrected M06‐2X functional and the 6–31++G(d,p) basis set, applying the polarizable continuum model (PCM) for the toluene solvent. Detailed information on the DFT calculations is provided in the Supporting Information.

### Mechanical Characterization of FD‐Ecoflex Composite

To analyze the mechanical properties of the FD‐Ecoflex composites, uniaxial tensile tests were conducted using a universal testing machine (Instron 3343, Instron, USA). All the composite samples for the mechanical characterization were fabricated with a 3D printed mold with a desired geometry, and the notch were made with a razor blade while controlling the notch size. The stress–strain curves were obtained with a strain rate of 10 mm min^−1^, and the crack propagation point was determined by monitoring with a camera for notched samples. The cyclic tensile tests were performed with a strain rate of 20 mm min^−1^.

### Fabrication and Characterization of Magnetically Actuated Soft Robot

NdFeB particle (80 µm; HKK solution, Korea) were used as a magnetic particle for fabrication of magnetically actuated soft robot. A resin with composition of Ecoflex‐0030: DPI‐HFP: FIA: NdFeB = 10: 2: 0.2: 3 (w/w) was placed in a mixing container and evenly mixed with a mixer (ARE‐310, THINKY, Japan) using the planetary centrifugal mixing mode, at a rotation speed of 2000 rpm for 2 min. Thereafter, the resin was poured onto a 3D printed mold of the desired geometry (length 20 mm; width 4 mm; thickness 1 mm). The resin was cured for 30 min in an oven at 70 °C. The cured composites were then wrapped around a cylindrical glass with a radius of 3 mm, and placed in a vibrating sample magnetometer (VSM‐7410, Lake shore, USA) inducing 3 T for magnetization. The magnetic field for soft robot actuation were induced using a commercial strong neodymium magnet (JL Magnet, Korea).

## Conflict of Interest

The authors declare no conflict of interest.

## Supporting information



Supporting Information

Supplemental Video 1

## Data Availability

The data that support the findings of this study are available from the corresponding author upon reasonable request.
