# Supplementary material for: UV‐Triggered Cascading Degradation of Silicone Elastomer via Self‐Fluoride Amplification
Source: Adv Sci (Weinh). 2025 Jul 11;12(38):e02056. doi: 10.1002/advs.202502056 (PMC12520503; doi:10.1002/advs.202502056)
Supplement: Supplementary file 1 — Supporting Information [file ADVS-12-e02056-s002.docx]

Supporting Information

Title UV-Triggered Cascading Degradation of Silicone Elastomer via Self Fluoride Amplification

Yoon-Nam Kim, Woojin Jeon, Min-Ha Oh, Hee-June Seo, Min Sang Kwon*, Seung-Kyun Kang*

**Note S1. Synthesis of fluoride ion amplifier**

**S1-1. Synthesis of methyl *p*-difluoromethylbenzoate**

Methyl p-difluoromethylbenzoate was synthesized according to the procedures previously reported literature.^[1]^ Dissolve methyl 4-formylbenzoate (1.4 g, 9.0 mmol) in diethylaminosulfur trifluoride (6.0 mL, 45 mmol) and stir for 12 h at RT. Dilute the reaction mixture with an excess of dichloromethane (80 mL), then slowly add water (80 mL). After phase separation, carefully wash the organic layer with saturated sodium carbonate aqueous solution. Dry the organic layer over sodium sulfate and filter off the solids. Concentrate the solution under reduced pressure. The residue was further purified by column chromatography on silica gel (ethyl acetate : hexane = 5 : 95 v/v) to obtain the pure product as a white solid (0.82 g, 49%). Data were in full agreement with those reported in literature.^[1]^

**S1-2. Synthesis of *p*-difluoromethylbenzoic acid**

*p*-difluoromethylbenzoic acid was synthesized according to the procedures previously reported literature.^[1]^ Dissolve Methyl p-difluoromethylbenzoate (1.0 g, 5.4 mmol) in 100 mL of diethyl ether and add potassium trimethylsilanolate (0.77 g, 6.0 mmol). Stir for 12 h at RT, then filter using filter paper. Wash the obtained white solid with hexane-ether (1:1 v/v). No further purification was required. Data were in full agreement with those reported in literature.^[1]^

**S1-3. Synthesis of (4-((tert-butyldimethylsilyl)oxy)phenyl)methanol**

(4-((tert-butyldimethylsilyl)oxy)phenyl)methanol was synthesized according to the procedures previously reported literature.^[2]^ Dissolve 4-hydroxybenzaldehyde (5.0 g, 41.0 mmol) and imidazole (5.58 g, 82.0 mmol) in anhydrous dichloromethane (90 mL). Under a nitrogen atmosphere, add tert-butyldimethylsilyl chloride (6.79 g, 45 mmol) slowly in an ice bath. Stir the mixture overnight at RT. Wash the reaction mixture with water and brine. After phase separation, dry the organic layer over sodium sulfate and concentrate under reduced pressure. Dissolve the crude product in methanol (40 mL) and add sodium borohydride (0.6 g, 15.0 mmol) slowly in an ice bath. Stir the mixture for 2 h at RT, then quench with brine. Evaporate the methanol under reduced pressure, and extract the organic layer with dichloromethane, water, and brine. Dry the organic layer over sodium sulfate and concentrate under reduced pressure. The residue was further purified by column chromatography on silica gel (ethyl acetate: hexane = 30:70 v/v) to obtain the product as an oily liquid (6.83 g, 70%). Data were in full agreement with those reported in literature.^[3]^

**S1-4. Synthesis of 4-((tert-butyldimethylsilyl)oxy)benzyl (4-(difluoromethyl)phenyl)-carbamate (FIA)**

4-((tert-butyldimethylsilyl)oxy)benzyl (4-(difluoromethyl)phenyl)carbamate was synthesized according to the procedures previously reported literature.^[1]^ Dissolve p-difluoromethylbenzoic acid (0.48 g, 2.3 mmol) in 1,2-dichloromethane (5.0 mL) and place it in an ice bath. Add thionyl chloride (0.42 mL, 5.8 mmol) dropwise to the reaction solution, followed by 8 drops of N,N-dimethylformamide. Stir the mixture at 100°C for 1 h. After cooling to RT, concentrate under reduced pressure. Dissolve the residue in acetone (2 mL) and place it in an ice bath. Add a solution of sodium azide (0.45 g, 6.9 mmol) in water (1.5 mL) dropwise while stirring in the ice bath for 1 h. Dilute the solution with ethyl acetate (20 mL) and dry the organic layer over sodium sulfate, then concentrate under reduced pressure. Dissolve the residue in toluene (5 mL) and stir at 100°C for 1 h. Cool the reaction mixture to RT and add (4-((tert-butyldimethylsilyl)oxy)phenyl)-methanol (0.55 g, 2.3 mmol) dissolved in toluene (1 mL) all at once. Stir the mixture at 100°C for 3 h. Concentrate the solution under reduced pressure and purify the residue by column chromatography on silica gel (ethyl acetate:hexane = 5:95 v/v) to obtain the product as a white solid (0.69 g, 72%). Data were in full agreement with those reported in literature.^[1]^

**Note S2. Density functional theory (DFT) calculation of Si-O backbone cleavage with F^-^**

DFT calculations were performed using the Gaussian16 program package with the M06-2X-D3/6–31++G(d,p) functional and basis set. The empirical dispersion correction was applied to account for dispersion interactions. The M06-2X method is well-suited for investigating thermochemistry and kinetics.^[4]^ Geometry optimizations and single-point energy calculations were conducted toluene solution employing the polarizable continuum model (PCM). All geometric optimization and frequency calculations were performed to confirm that the structures represent true minima and to obtain the free energy at 423.15 K. In the transition state, a single imaginary frequency was identified, and intrinsic reaction coordinate (IRC) calculations were carried out to verify the nature of the transition state. For computational efficiency, the terminal groups of the PDMS polymer structure were replaced with methoxy groups. The Gibbs free energy with relevant standard state reference was obtained from following relations,^[5]^ *G^0^ (423.15 K, 1 M) = G^0^* (423.15 K, 1 atm) + 2.98 kcal/mol.*

**Figure S1** ^1^H NMR data of methyl *p*-difluoromethylbenzoate at RT (400 MHz, CDCl_3_). H NMR (400 MHz, CDCl_3_) δ 8.12 (d, *J* = 8.1 Hz, 2H), 7.58 (d, *J* = 8.3 Hz, 2H), 6.69 (t, *J* = 56.1 Hz, 1H), 3.94 (s, 3H)

**Figure S2** ^1^H NMR data of *p*-difluoromethylbenzoic acid at RT (400 MHz, CD_3­_OD). ^1^H NMR (400 MHz, CD_3­_OD) δ 8.04 (d, *J* = 7.9 Hz, 2H), 7.58 – 7.49 (m, 2H), 6.79 (t, *J* = 56.3 Hz, 1H).

**Figure S3** ^1^H NMR data of (4-((tert-butyldimethylsilyl)oxy)phenyl)methanol at RT (400 MHz, CDCl_3_). ^1^H NMR (400 MHz, CDCl_3_) δ 7.20 (d, *J* = 8.6 Hz, 2H), 6.84 – 6.78 (m, 2H), 4.56 (d, *J* = 3.8 Hz, 2H), 1.99 (d, *J* = 10.1 Hz, 1H), 0.98 (s, 9H), 0.19 (s, 6H).

**Figure S4** ^1^H NMR data of 4-((tert-butyldimethylsilyl)oxy)benzyl (4-(difluoromethyl)-phenyl)carbamate at RT (500 MHz, Acetone-d_6_). ^1^H NMR (500 MHz, Acetone-d_6_) δ 7.71 (d, *J* = 8.3 Hz, 2H), 7.52 (d, *J* = 8.3 Hz, 2H), 7.37 – 7.32 (m, 2H), 6.96 – 6.70 (m, 3H), 5.11 (s, 2H), 0.99 (s, 9H), 0.22 (s, 6H)


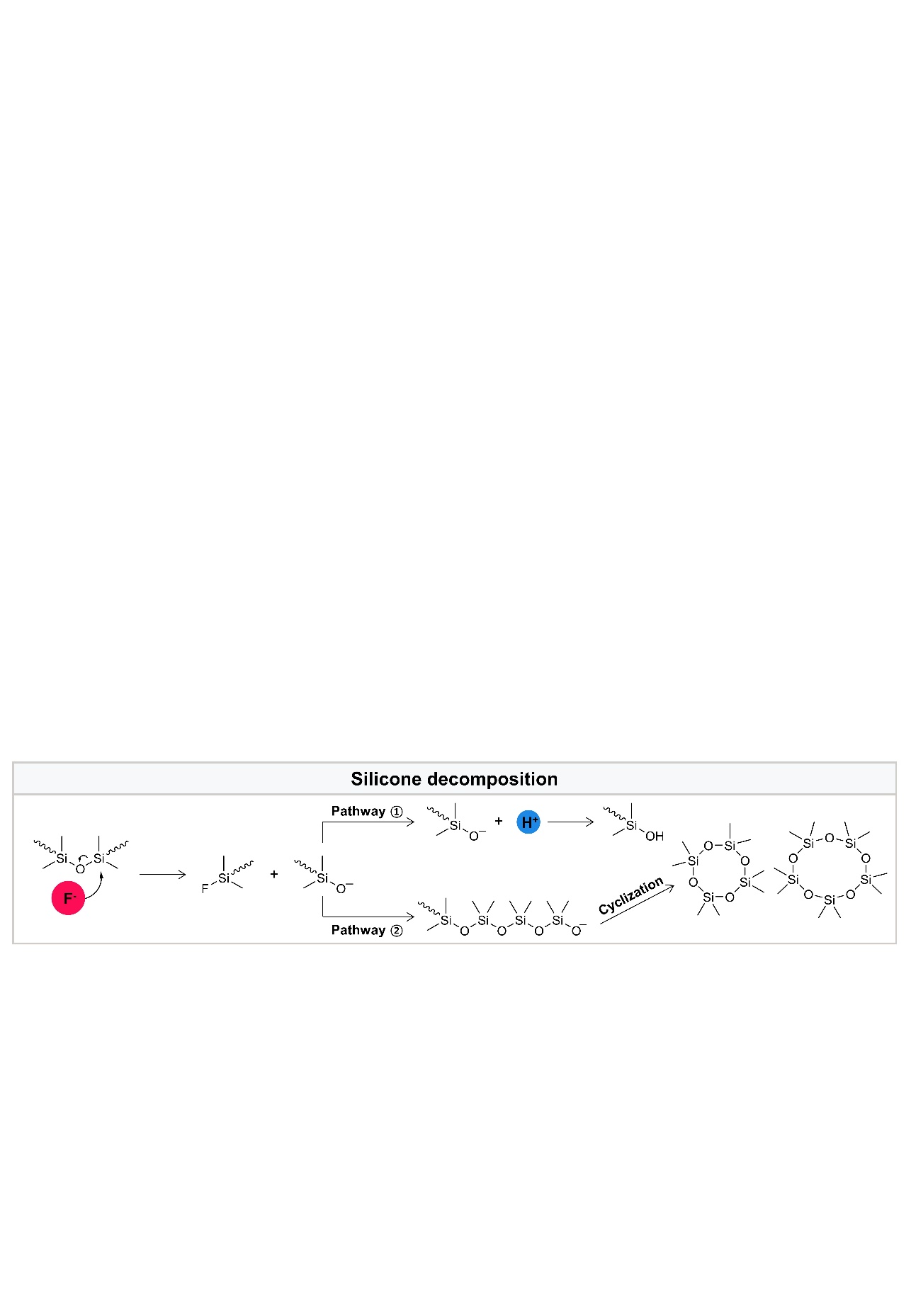


**Figure S5.** Reaction scheme for the degradation of silicone via F⁻, generating Si-F bond and Si-OH bond with other siloxane fragments.
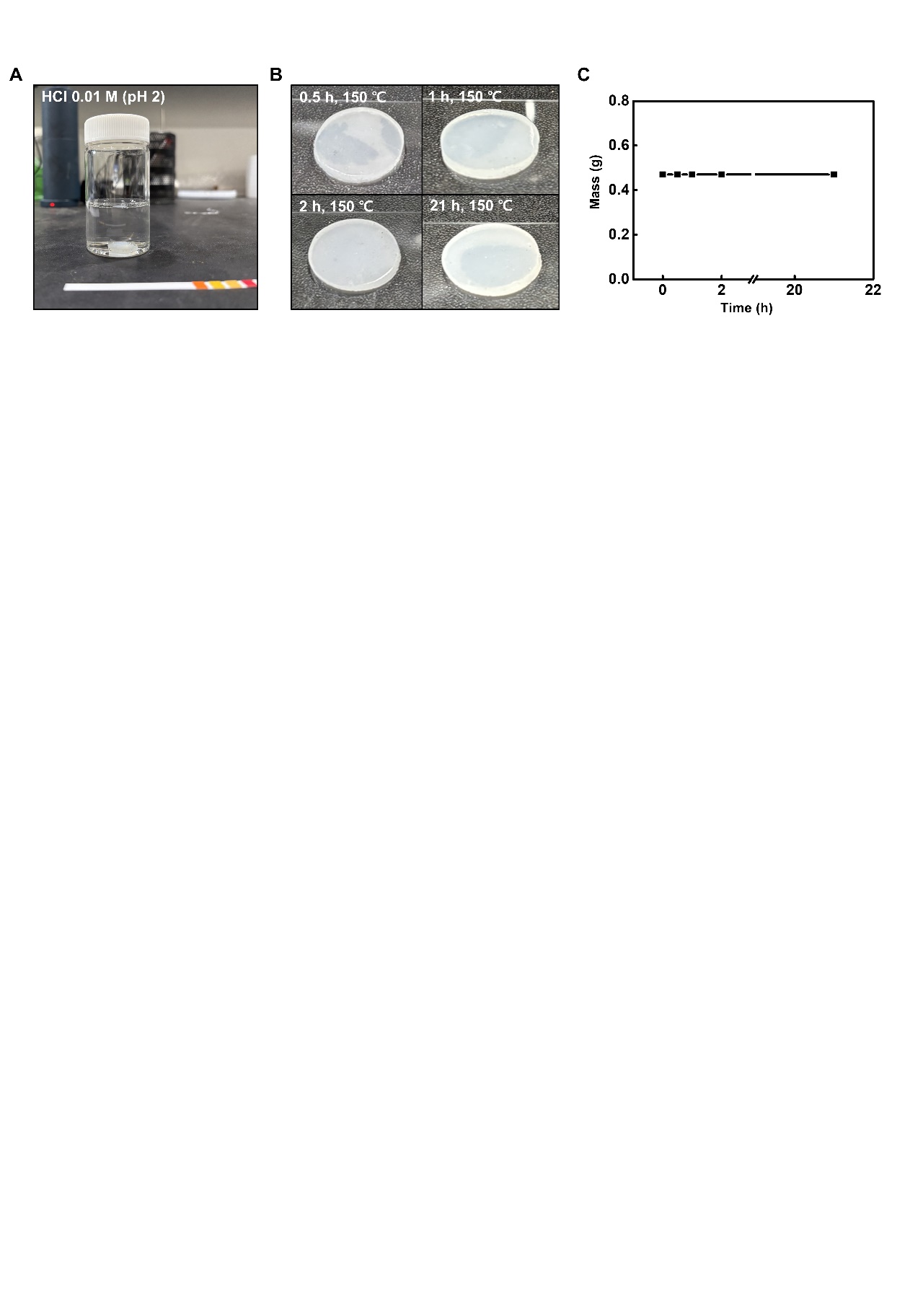


**Figure S6.** Stability of bare silicone elastomer (Ecoflex 0030) with acidic condition (pH 2, HCl) (A) Experimental set-up of stability of silicone elastomer under acidic condition (B) time-lapse image of silicone elastomer under acidic (pH 2) and high temperature (150 ℃) condition (C) Mass change of the silicone elastomer under acidic (pH 2) and high temperature (150 ℃) condition.

**
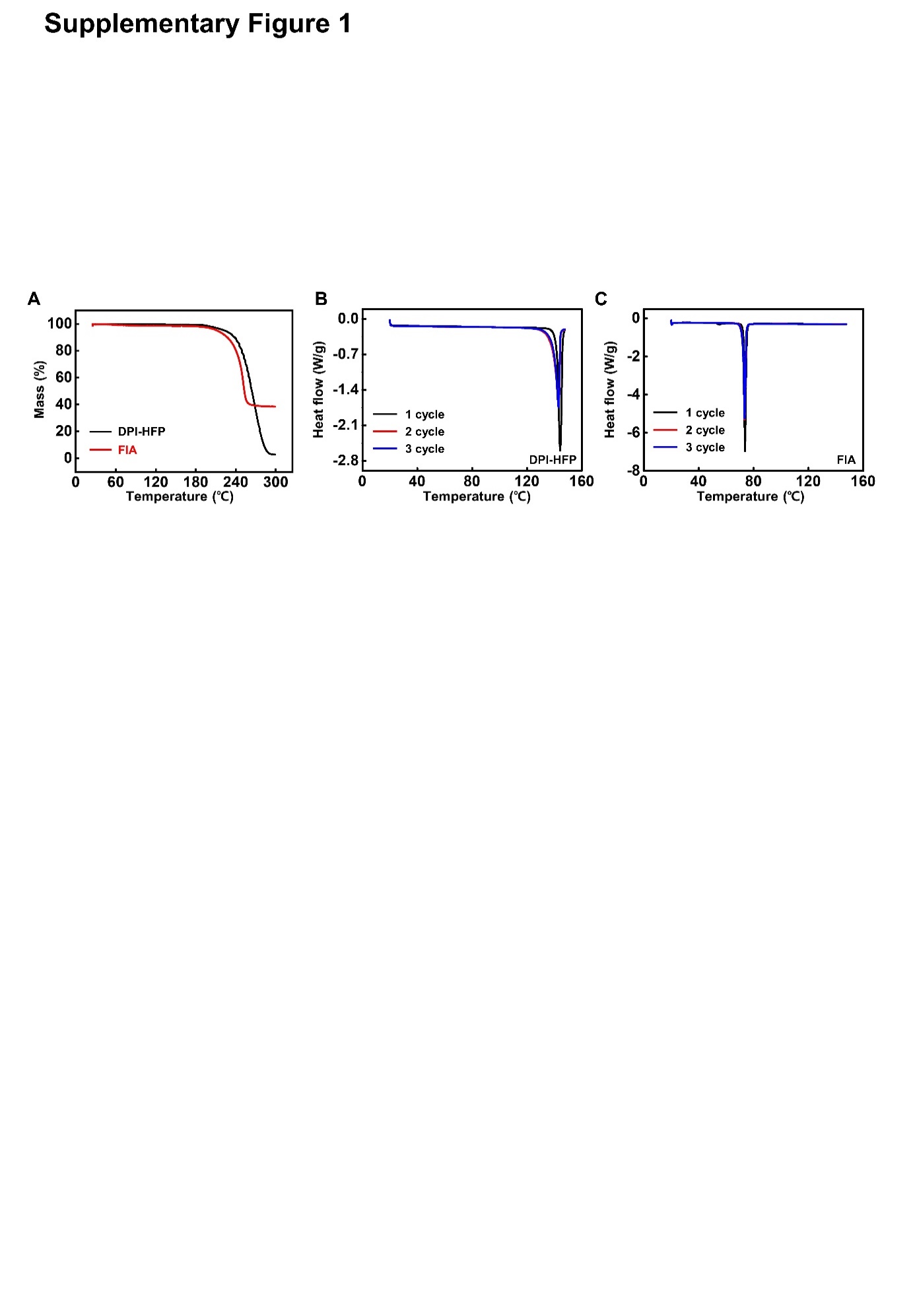
**

**Figure S7.** (a) TGA data of DPI-HFP (black) and FIA (red) ranging from 25 ℃ to 300 ℃ (heating rate 10 ℃ min^-1^) with onset of mass loss at 239.2 ℃ (DPI-HFP), and 239.9 ℃ (FIA), respectively. Cyclic DSC analysis of (b) DPI-HFP and (c) FIA from 25 ℃ to 150 ℃ with heating and cooling rate of 10 ℃ min^-1^. Heat flow of the 1^st^ cycle (black), 2^nd^ cycle (red) and 3^rd^ cycle (blue) showing the same peak temperature of 144.4 ℃ (DPI-HFP) and 73.9 ℃ (FIA) due to physical melting without any chemical decomposition.

**
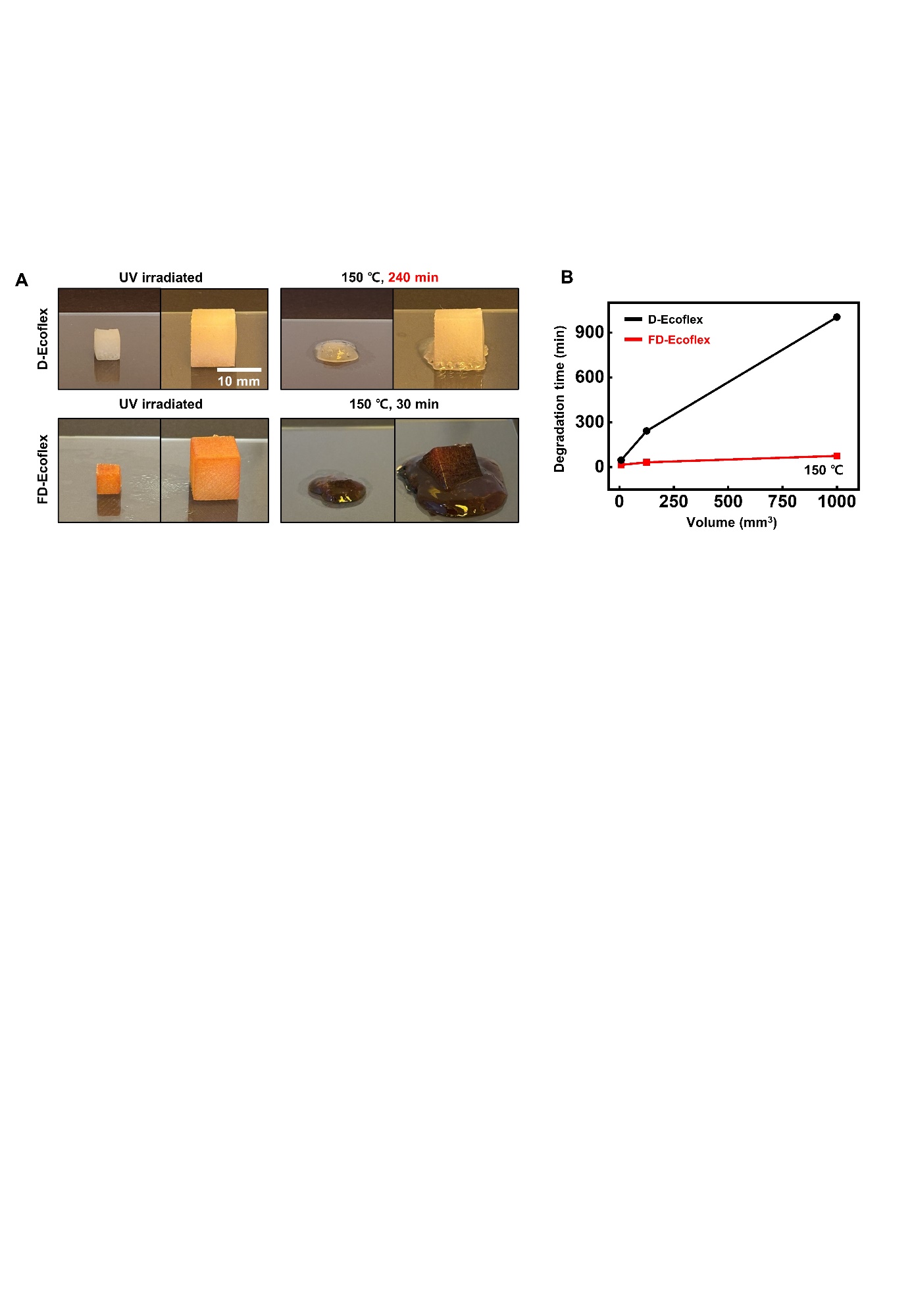
**

**Figure S8.** (a) Image of D-Ecoflex (top) and FD-Ecoflex (bottom) with the volume of 125 mm^3^ and 1000 mm^3^ after 30 min UV irradiation (left) and heat exposure at 150 ℃ (right). Image acquired after 240 min heating for D-Ecoflex and after 30 min heating for FD-Ecoflex. (b) Degradation time at 150 ℃ with varying volume of the D-Ecoflex (black) and FD-Ecoflex (red). Time of complete degradation roughly estimated at the point where the composite was no longer visible to the naked eye.


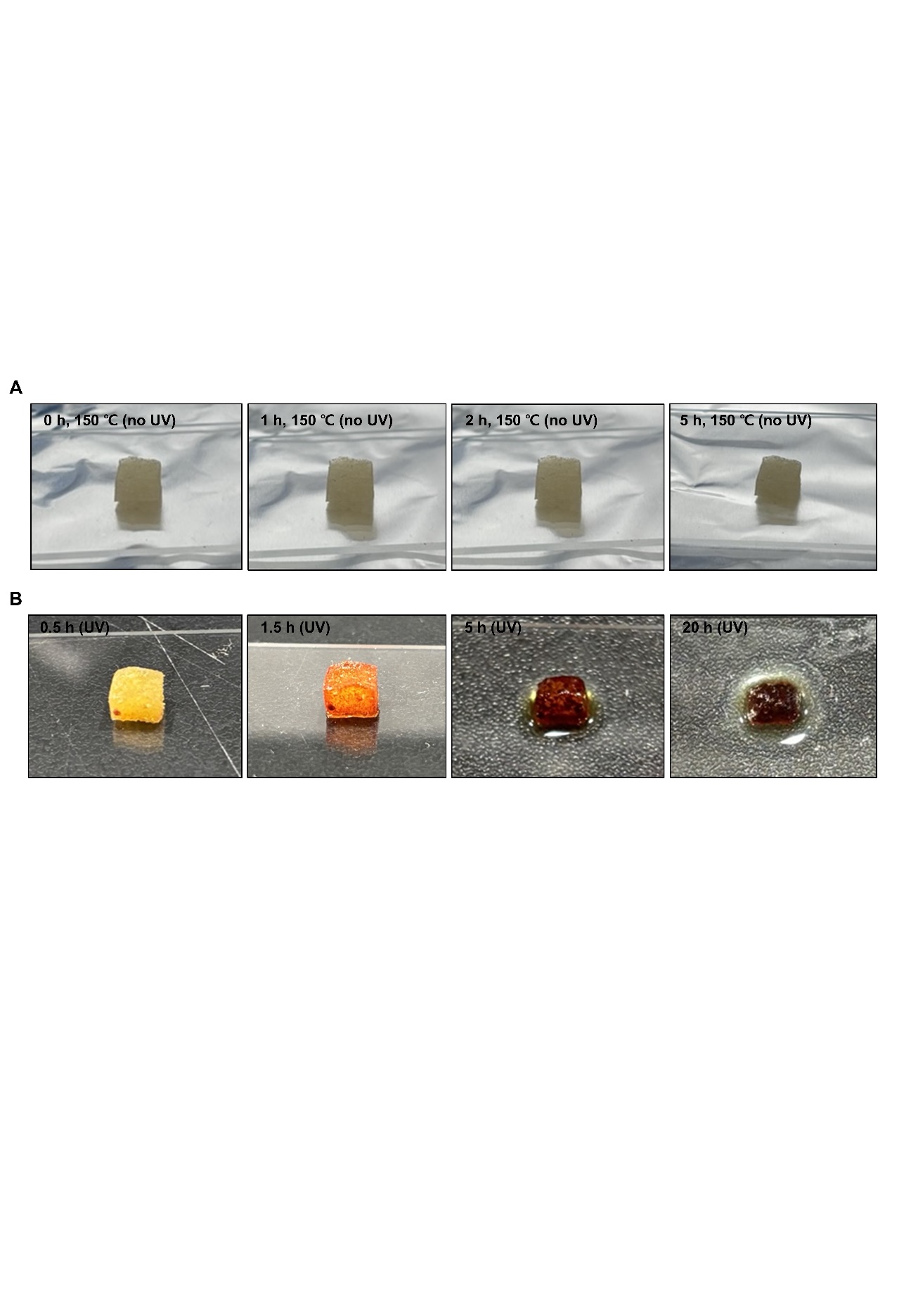
**Figure S9.** Time-lapse image of FD-Ecoflex with (A) only heat exposure (150 ℃) and with (B) only UV exposure at room temperature. Complete disappearance of initial shape after 20 hours of UV exposure.


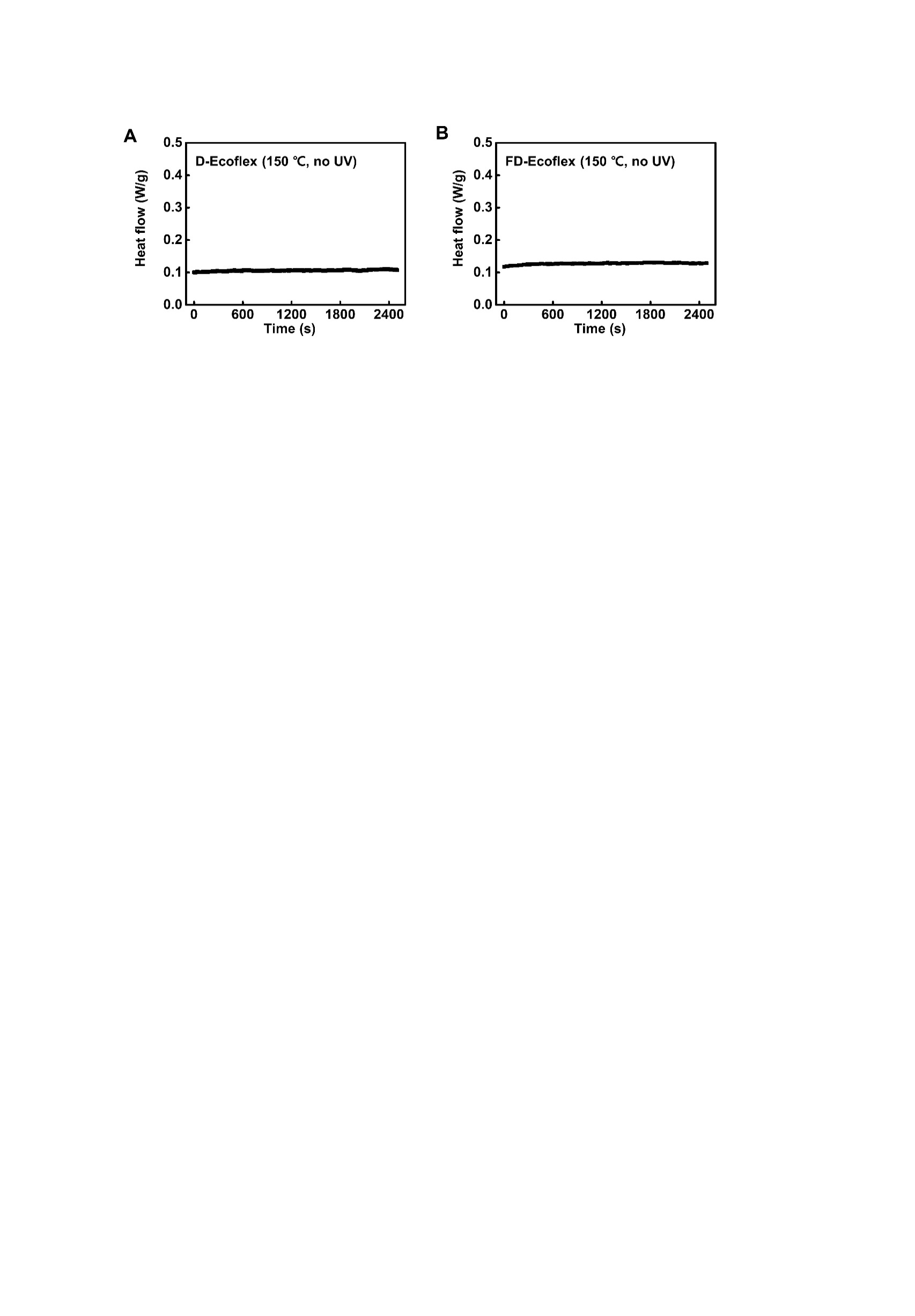


**Figure S10.** Isothermal (150 °C) differential scanning calorimetry (DSC) thermograms of (A) D-Ecoflex and (B) FD-Ecoflex without UV pretreatment.


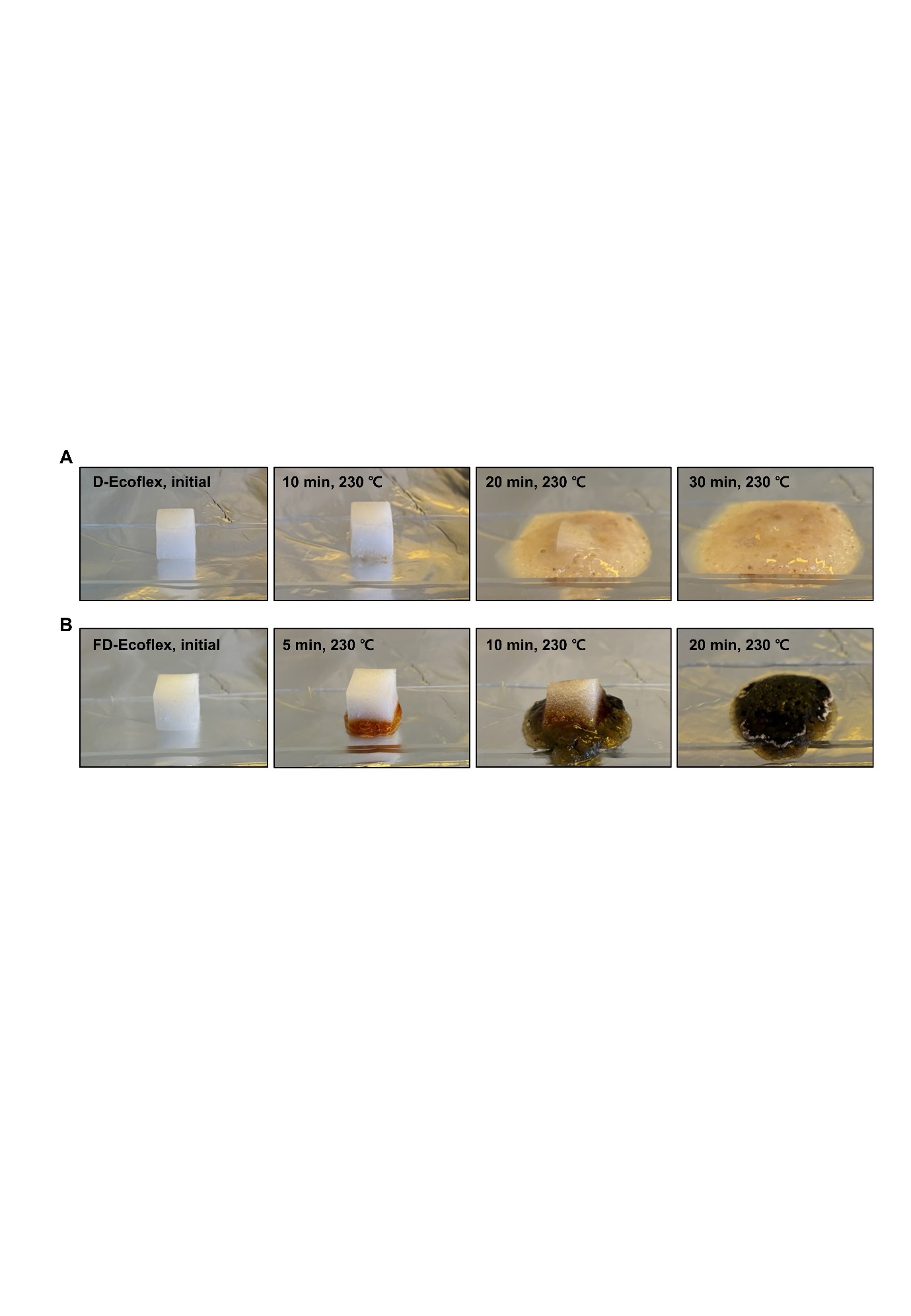


**Figure S11.** Time-lapse image of (A) D-Ecoflex and (B) FD-Ecoflex at extremely high temperature (230 ℃) without prior UV-trigger, near the thermal decomposition temperature of DPI-HFP and FIA. Both samples losing thermal stability over 230 ℃.


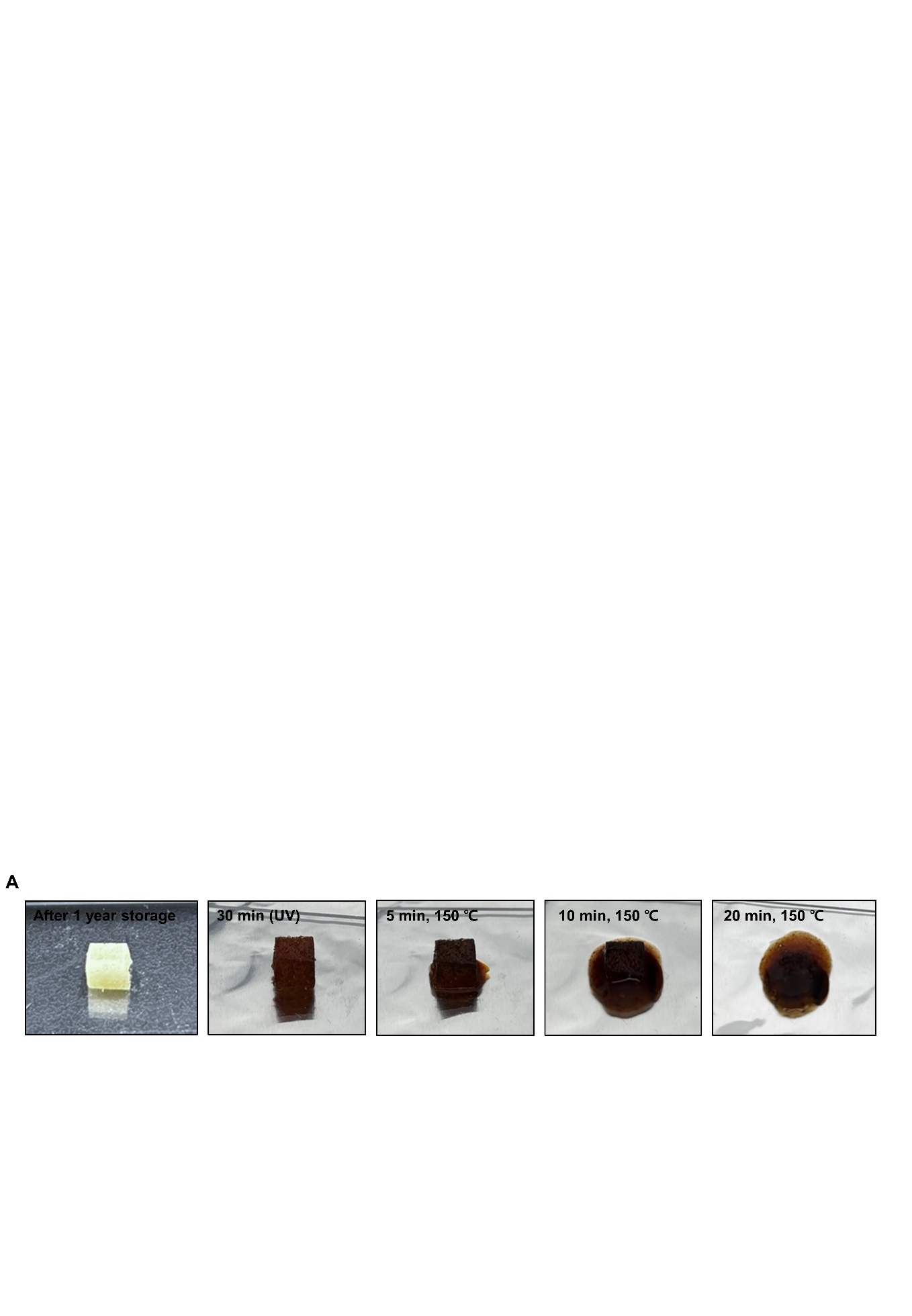


**Figure S12.** Sequential image of UV-triggered FD-Ecoflex degradation fabricated 1 year prior stored at ambient condition. Dark colorimetric shift with UV-trigger and complete degradation within 20 minutes of heating (150 °C).

**Figure S13** ^1^H NMR data before and after 30 minutes of UV irradiation with DPI-HFP + FIA (10:1 molar ratio) at RT (500 MHz, Acetone-d_6_). The blue line represents the data after 30 minutes of UV irradiation, while the yellow line represents the data before UV irradiation.

**Figure S14** ^1^H NMR data before and after 30 minutes of UV irradiation with FIA at RT (500 MHz, Acetone-d_6_). The blue line represents the data after 30 minutes of UV irradiation, while the yellow line represents the data before UV irradiation.

**
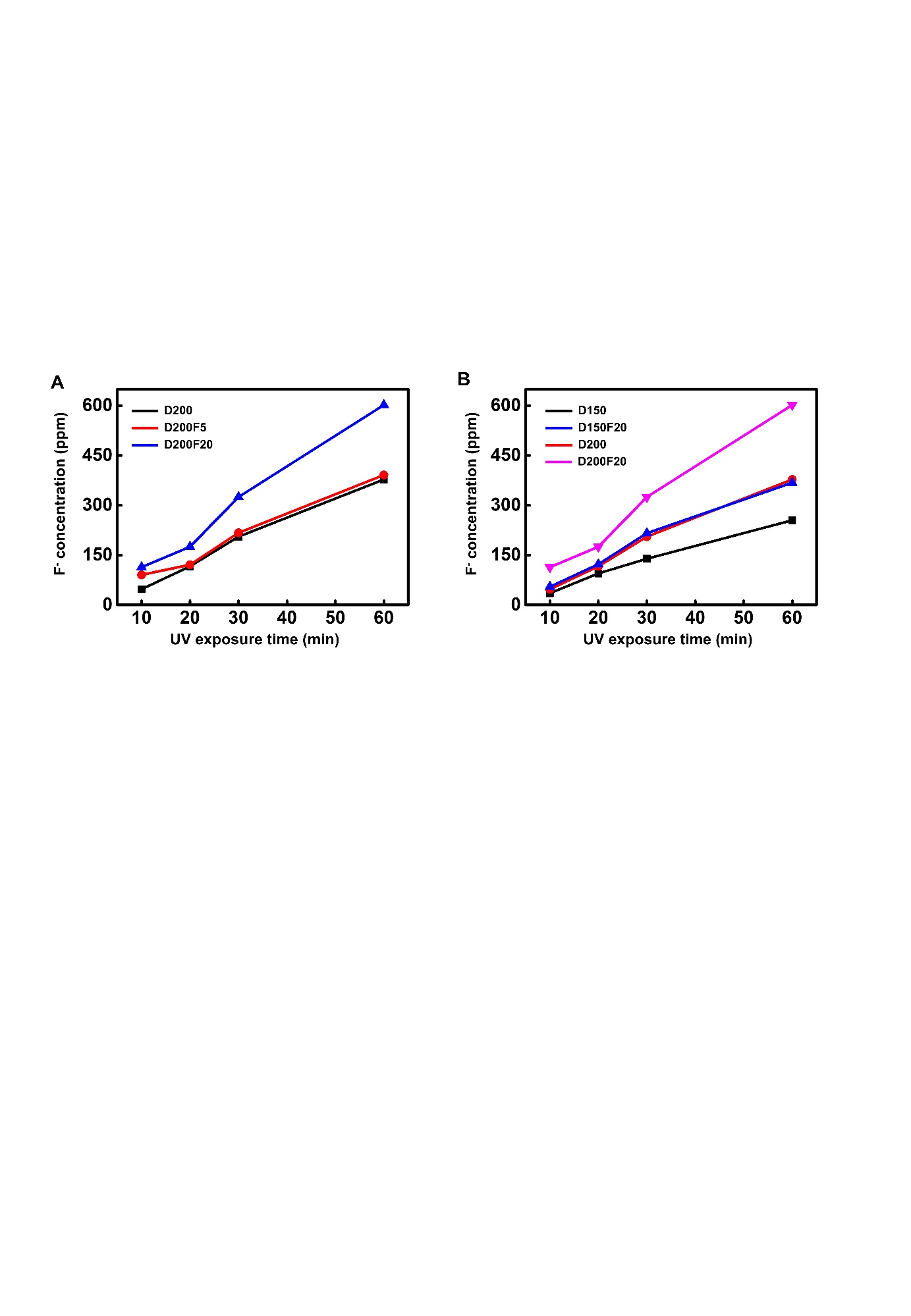
**

**Figure S15** F^–^ concentration analysis with varying DPI-HFP and FIA mass ratio (A) DPI-HFP 200 mg (D200) with varying FIA mass DPI-HFP 200 mg + FIA 5 mg (D200F5) and DPI-HFP 200 mg + FIA 20 mg (D200F20), and (B) varying DPI-HFP with and without FIA (D150, D200, D150F20, D200F20), respectively.

**
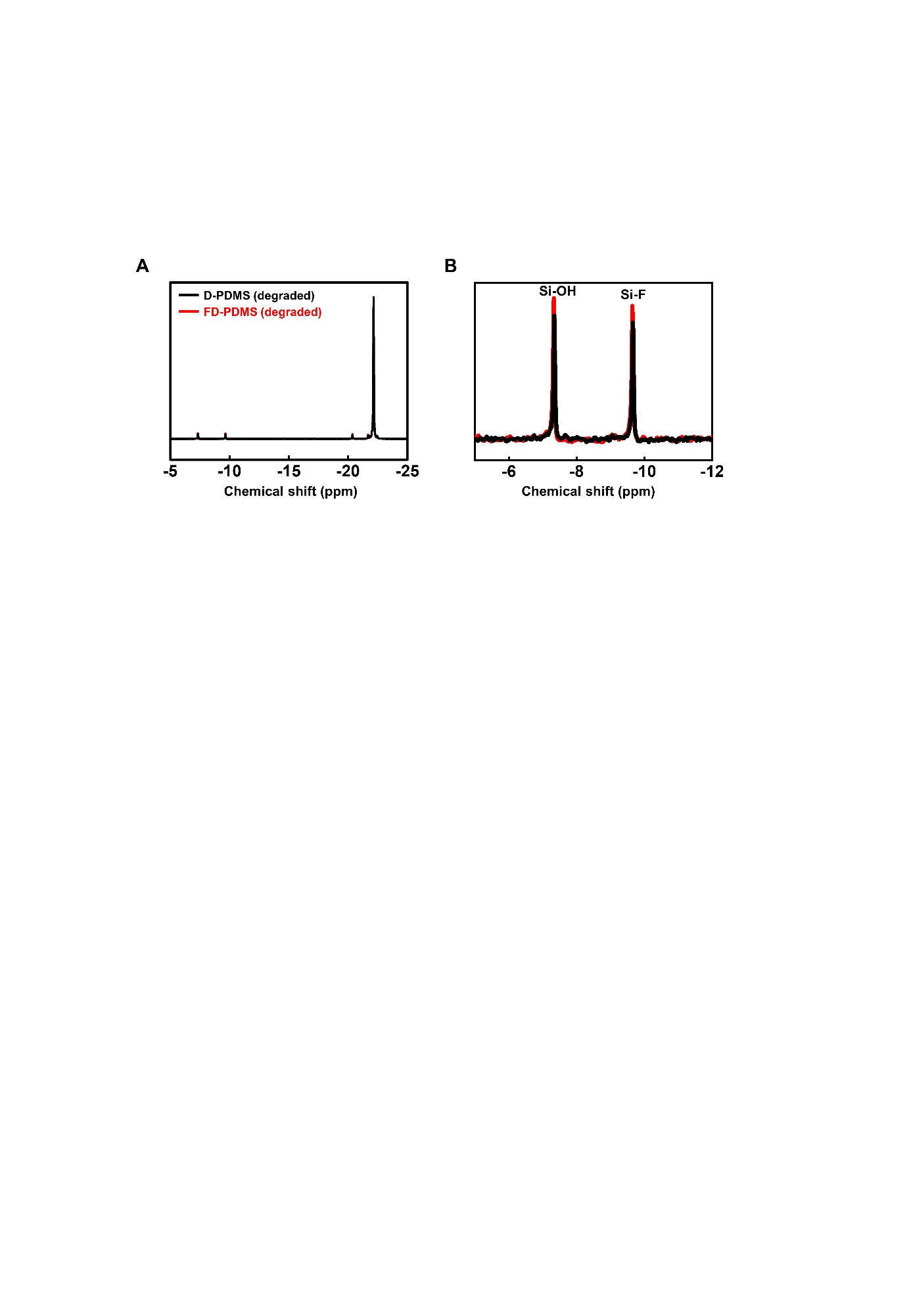
**

**Figure S16.** (a) ^29^Si-NMR analysis data of UV-triggered linear PDMS (M.W 4800) with DPI-HFP (D-PDMS; black) and with FIA + DPI-HFP (FD-PDMS; red). (b) Magnified view from -5 ppm to -12 ppm showing increased Si-OH (-7 ppm) and Si-F (-9 ppm) peak with FD-PDMS.


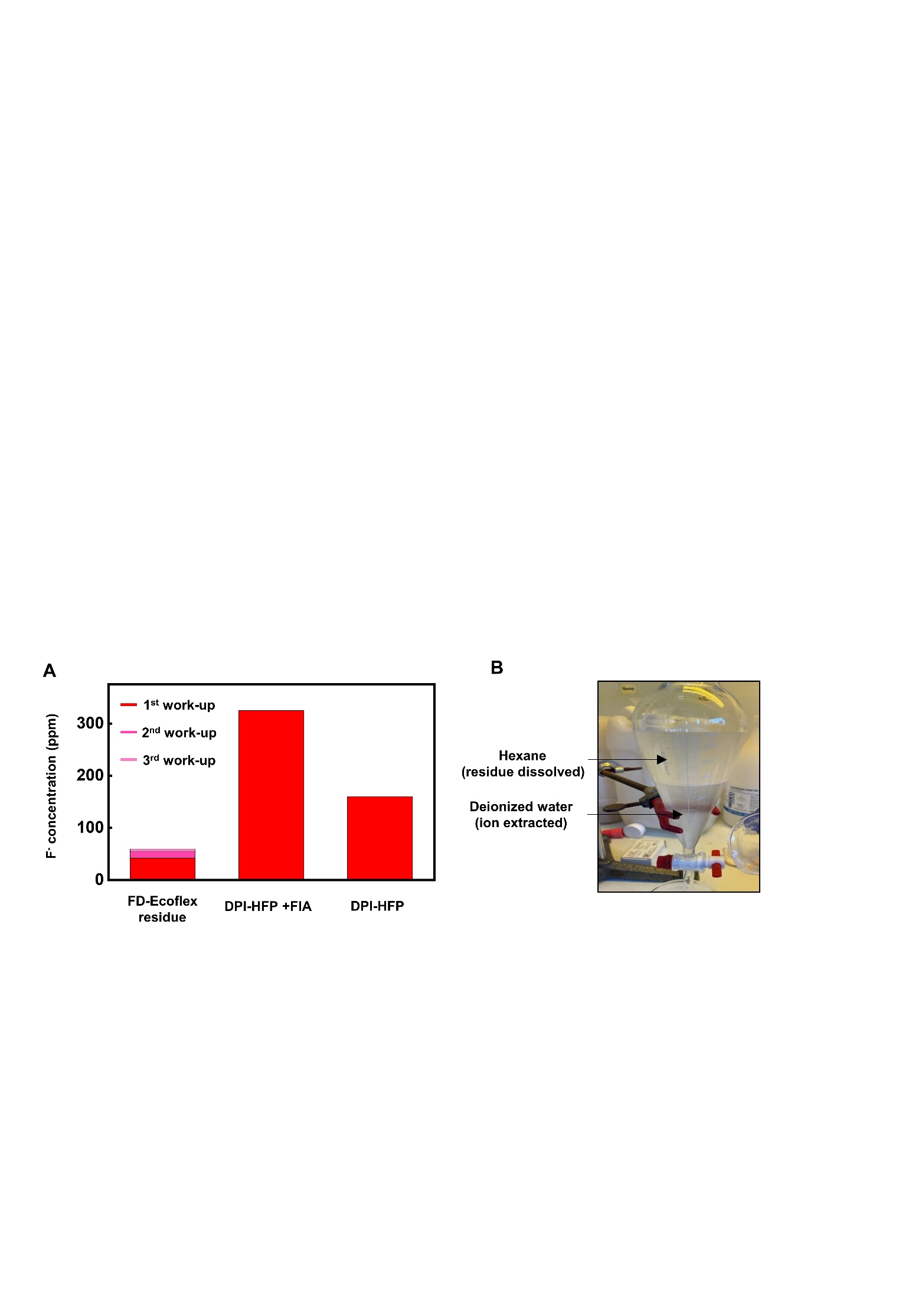


**Figure S17.** (a) ^29^Si-NMR analysis data of UV-triggered linear PDMS (M.W 4800) with DPI-HFP (D-PDMS; black) and with FIA + DPI-HFP (FD-PDMS; red). (b) Magnified view from -5 ppm to -12 ppm showing increased Si-OH (-7 ppm) and Si-F (-9 ppm) peak with FD-PDMS.


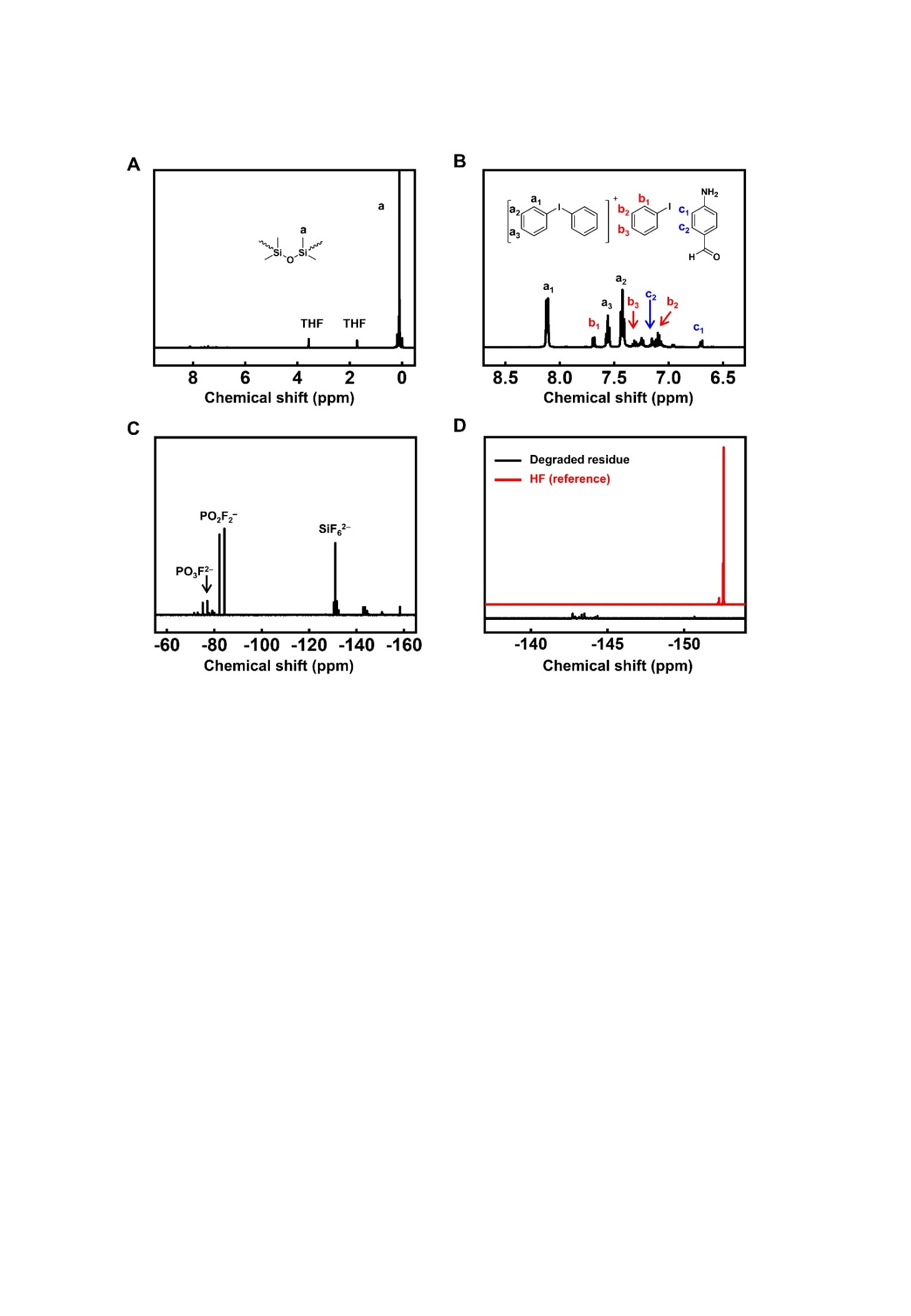


**Figure S18.** (a) ^29^Si-NMR analysis data of UV-triggered linear PDMS (M.W 4800) with DPI-HFP (D-PDMS; black) and with FIA + DPI-HFP (FD-PDMS; red). (b) Magnified view from -5 ppm to -12 ppm showing increased Si-OH (-7 ppm) and Si-F (-9 ppm) peak with FD-PDMS.

**Figure S19.** The proposed mechanism pathway for Si–O bond cleavage by HF (top) and the energy profile calculated by DFT for the reaction pathway (bottom) are described.

**
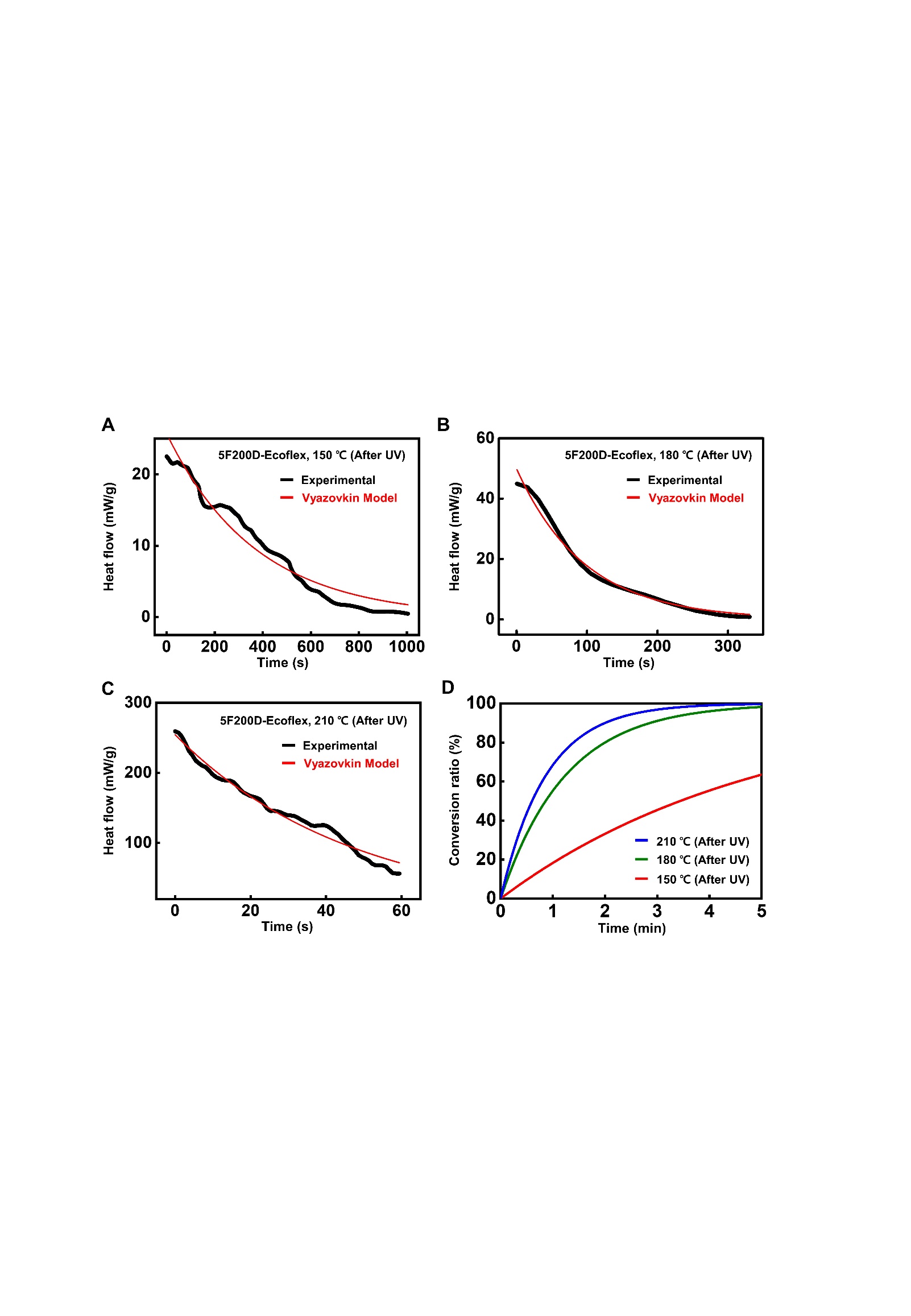
**

**Figure S20.** Degradation thermodynamics analysis of FD-Ecoflex with reduced FIA mass ratio (FIA 0.5 wt%, DPI-HFP 20 wt%; 5F200D-Ecoflex). Differential scanning calorimetry (DSC) analysis of 5F200D-Ecoflex with varying temperature (A) 150 °C, (B) 180 °C and (C) 210 °C, respectively. Reaction coefficient (*k*) derived with Vyazovkin model showing the value of 0.0027 (150 °C), 0.0103 (180 °C) and 0.0213 (210 °C), all decreased compared to FD-Ecoflex (FIA 2 wt%, DPI-HFP 20 wt%). (D) Extent of phase conversion for 5F200D-Ecoflex with varying temperature.


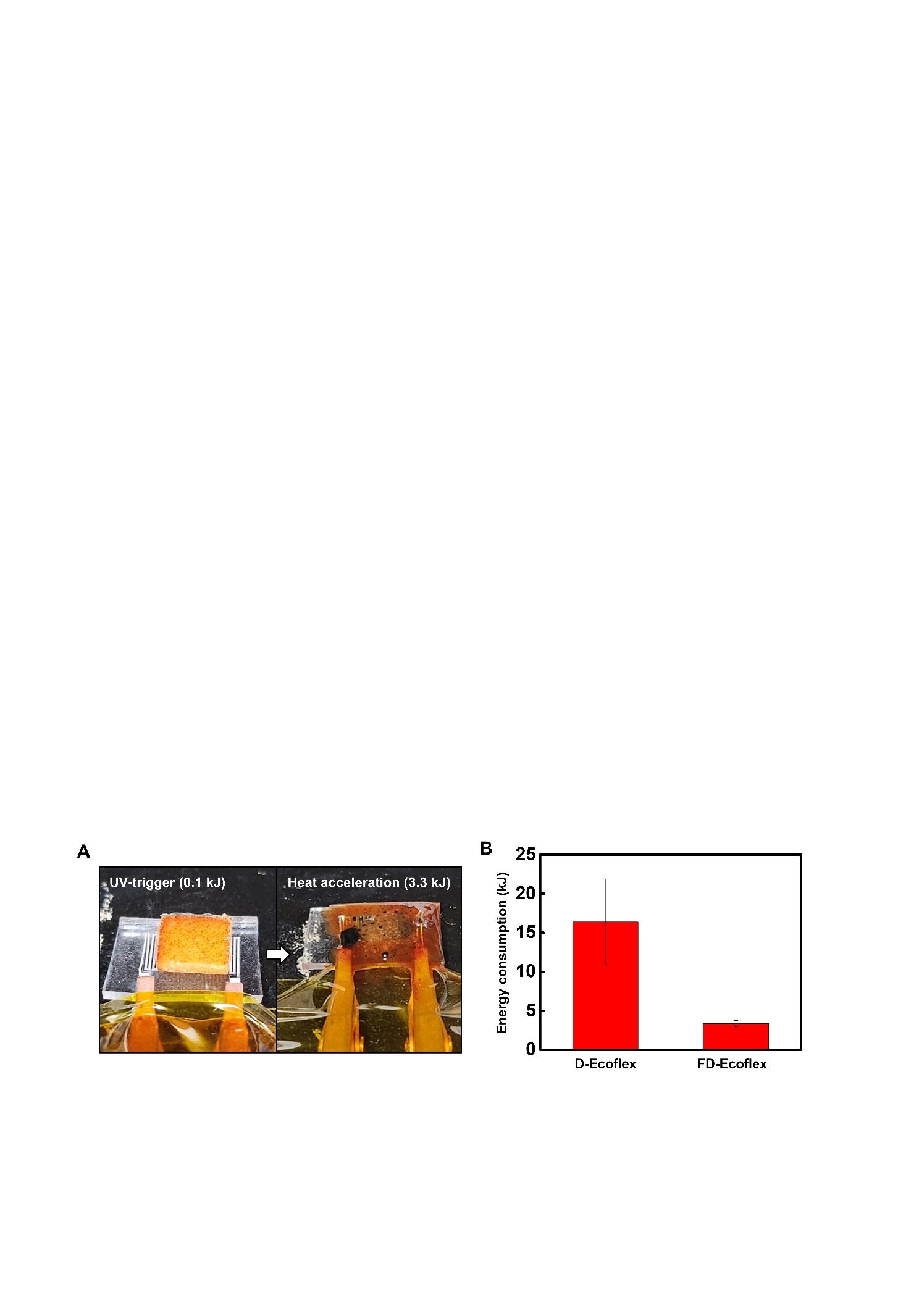


**Figure S21.** Energy consumption analysis for complete degradation of UV-triggered FD-Ecoflex (A) sequential image of FD-Ecoflex degradation (B) Energy consumption comparison between D-Ecoflex and FD-Ecoflex.


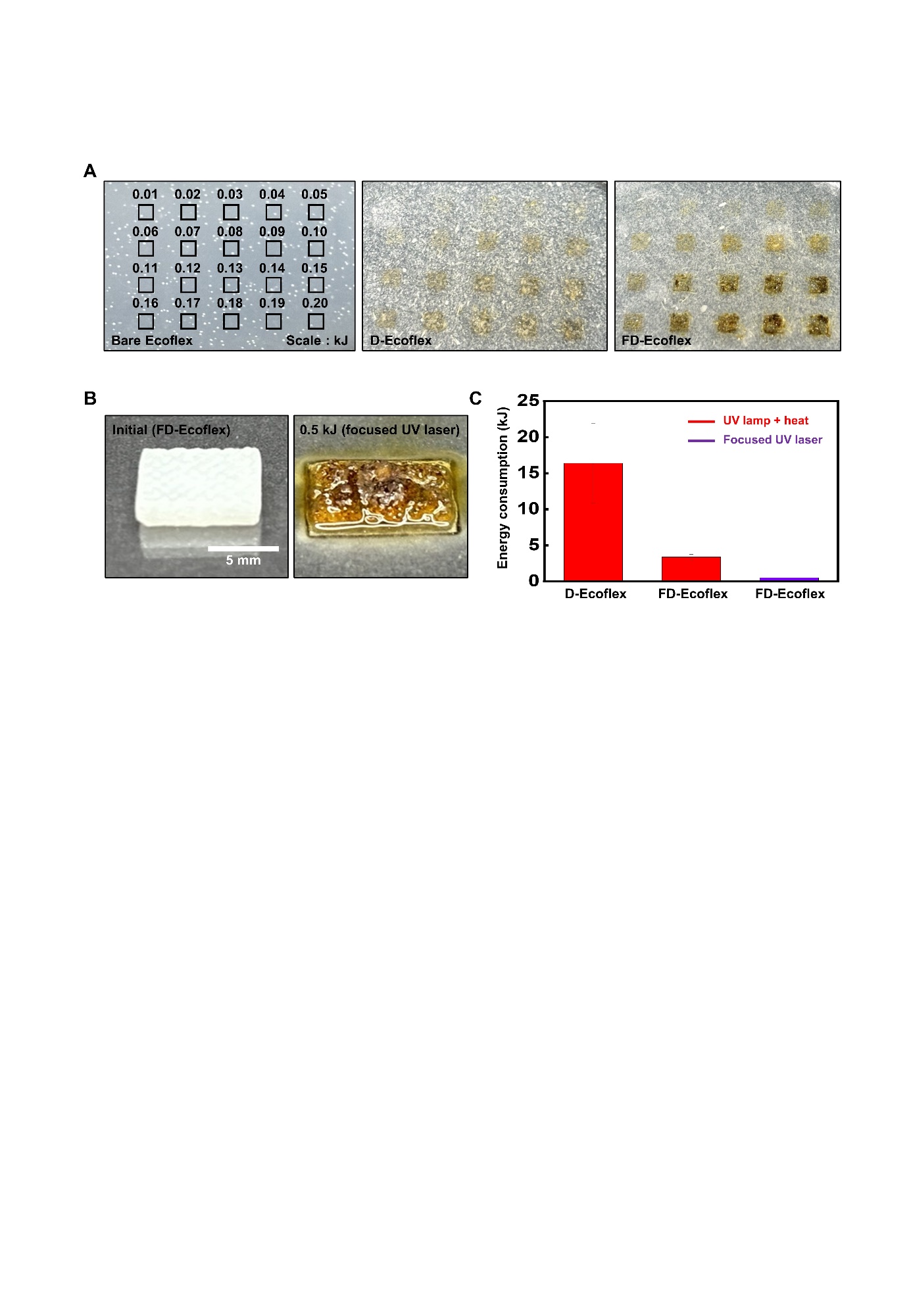


**Figure S22.** Degradation behavior and energy consumption for complete degradation with focused UV laser (125 mW/cm^2^). (A) Degradation behavior comparison with focused UV laser irradiation at energy doses varying from 0.01 kJ to 0.2 kJ for Bare Ecoflex (left), D-Ecoflex (middle), and FD-Ecoflex (right), respectively. FD-Ecoflex showing bulk degradation with over 0.17 kJ. (B) Image of FD-Ecoflex bulk degradation with 0.5 kJ of focused UV laser. (C) Comparison of energy consumption for complete degradation. FD-Ecoflex with greatly reduced energy consumption with focused UV laser, while D-Ecoflex was not possible to degrade with only focused UV laser.

**Supplementary References**

[1] M. S. Baker, S. T. Phillips, *J. Am. Chem. Soc.* **2011**, *133*, 5170–5173.

[2] X. Xu, W. Chen, M. Yang, X. J. Liu, F. Wang, R. Q. Yu, J. H. Jiang, *Talanta* **2019**, *204*, 655–662.

[3] Y. H. Chen, W. C. Chien, D. C. Lee, K. T. Tan, *Anal. Chem.* **2019**, *91*, 12461–12467.

[4] Y. Zhao, D. G. Truhlar, *Theor. Chem. Acc.* **2008**, *120*, 215–241.

[5] H. G. Korth, P. Mulder, *J. Org. Chem.* **2020**, *85*, 2560–2574.
